# Iodine-mediated nucleation and particle growth: laboratory measurements of IO production and its implications

**DOI:** 10.1039/d6ra01191h

**Published:** 2026-05-26

**Authors:** Yuhao Yan, Shanshan Wang, Zhiwen Jiang, Chuanqi Gu, Shuyao Xiang, Alfonso Saiz-Lopez, Bin Zhou

**Affiliations:** a Shanghai Key Laboratory of Air Quality and Environmental Health, Department of Environmental Science and Engineering, Fudan University Shanghai 200433 China; b Institute of Eco-Chongming (IEC) Shanghai 202151 China; c Department of Atmospheric Chemistry and Climate, Institute of Physical Chemistry Blas Cabrera, CSIC Madrid 28006 Spain; d Institute of Atmospheric Sciences, Fudan University Shanghai 200433 China

## Abstract

Reactive iodine chemistry significantly influences atmospheric oxidation and new particle formation. The detailed chemical mechanism linking iodine monoxide (IO) radicals to particle nucleation and growth, however, remains insufficiently constrained. Here, we designed a dual-optical cell Differential Optical Absorption Spectroscopy (DOAS) system to generate and measure IO. Combined with laboratory measurements and an observation-constrained chemical model, we revealed the direct correlation between IO and particle formation under controlled O_3_ and I_2_ levels, and further provided modeling insights into the potential roles of higher iodine oxides and iodic acid (HIO_3_) in aerosol dynamics. Results showed that IO although not a nucleating species, can promote the formation of intermediates, driving nucleation and size-dependent growth. Observation-constrained model simulations revealed that HIO_3_ concentrations exhibited a power-law dependence on particle growth rates (GR) across different size bins. Notably, under dry laboratory conditions (RH ∼5.5%), HIO_3_ may remain an important contributor to particle growth, while higher iodine oxides like I_3_O_7_, played minor roles. An increase in O_3_ from 10 to 50 ppbv was associated with enhanced HIO_3_ production and a more than 20% increase in GR for the 1.8–3.2 nm size range. These results suggest that, under dry iodine-rich conditions, HIO_3_ may be relevant to particle growth, although the extent of its atmospheric importance will depend on local precursor levels and environmental conditions. In addition, variations in ambient O_3_ may influence HIO_3_ formation and, consequently, iodine-mediated particle growth. These findings provide experimental and modeling evidence that nonlinear O_3_-iodine interactions may need to be considered for accurately evaluating the impact of iodine chemistry on aerosol loading and regional air quality.

## Abstract 2

Laboratory measurements and an observation-constrained model reveal the response of iodine-mediated particle formation and growth to initial IO levels, with HIO_3_ playing a more dominant role.

## Graphical abstract

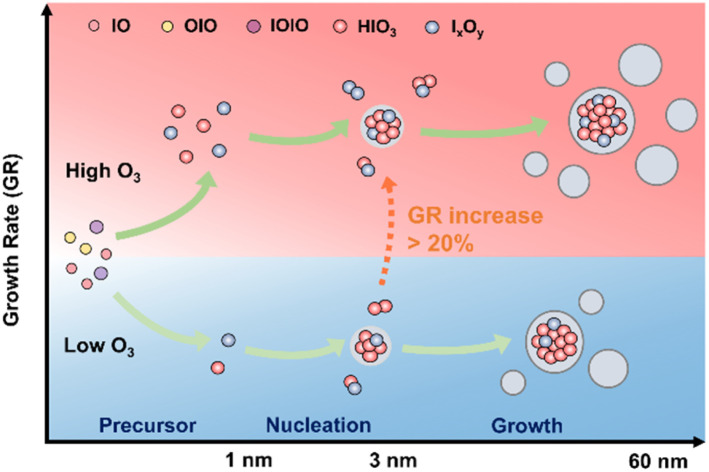

## 1. Introduction

The role of iodine in atmospheric chemistry has received increasing attention during the last few decades.^1^ Reactive iodine species (RIS) influence atmospheric oxidative capacity *via* a series of photochemical reactions, including catalytic destruction of ozone, alterations in OH radical production and NO/NO_2_ ratio,^2–6^ and exert an indirect effect on climate.^7^ Additionally, studies have shown that iodine plays a critical role in the formation of ultrafine aerosol particles.^8–11^ The primary sources of atmospheric iodine are the ocean, polar regions, and salt lakes.^12^ Molecular iodine (I_2_) and hypoiodous acid (HOI) are emitted from the ocean through the reactions of atmospheric O_3_ with aqueous iodide (I^−^).^13^ The main formation of iodine monoxide radical (IO) is the reaction of O_3_ with atomic iodine formed *via* photolysis of I_2_ (or HOI, CH_3_I and CH_2_I_2_).^12^ Then IO undergoes self-reaction to produce iodine dioxide (OIO) and I_2_O_2_.^14^ Higher iodine oxides (I_*x*_O_*y*_) and iodic acid (HIO_3_) can be formed through subsequent reactions involving IO, OIO and I_2_O_2_,^15,16^ which are considered as key species in the formation and growth of iodine particles.^17–20^

Since the first detection of IO in the marine boundary layer (MBL) at Mace Head, Ireland,^21^ measurements of RIS have been conducted in different regions worldwide, including MBL,^22–27^ the Arctic and Antarctic boundary layer,^9,28,29^ salt lakes,^30^ the lower and upper free troposphere,^31–33^ and the lower stratosphere.^34^ Despite strong correlations between iodine-containing species and particle formation and growth observed globally,^8,35–38^ field studies have often focused only on correlating I_2_/IO bursts or HIO_3_ levels with new particle formation (NPF) events, but few have captured the complete sequence of processes from IO formation to higher iodine oxides and HIO_3_ generation, and ultimately to iodine particle formation and growth.

Laboratory studies on the mechanisms of iodine particle formation and growth have been carried out under different environmental variables. In early experiments, OIO and I_2_O_*y*_ are considered to be the primary drivers of iodine particle formation and growth.^39–41^ A more recent laboratory study has suggested that higher iodine oxides are the major species responsible for the growth and composition of iodine particles.^42^ Recent field and environmental studies have reported a mechanism for HIO_3_ nucleation.^17,18,43^ He *et al.*^18^ concluded that HIO_3_ plays a dominant role in the iodine particle formation and growth at ambient vapor concentrations, which is supported by laboratory experiments. Studies using flow tube indicate that the precursors of iodine particles might be the I_2_O_*y*_·HIO_3_ clusters at high water and low iodine concentrations, and I_*x*_O_*y*_ clusters are more likely to be the precursors under dry conditions.^20^ In addition, some studies have reported that iodous acid (HIO_2_), methanesulfonic acid (MSA), and organic vapors can promote iodine-driven nucleation and particle growth.^44–46^ To conclude, the key precursors and reaction pathways involved in the formation and growth of iodine particles require further exploration. Moreover, there is a lack of integrated discussion linking IO oxidation chemistry with subsequent particle nucleation and growth *via* intermediate species such as higher iodine oxides and HIO_3_.

Here, we conducted experiments using a newly designed dual-optical cell Differential Optical Absorption Spectroscopy (DOAS) system to generate and measure IO, and investigated the gas-to-particle conversion process with controlled environmental variables. In the laboratory, IO radicals were generated through the photolysis of mixed I_2_ and O_3_ at varying concentrations, and their impacts on the particle number size distribution were evaluated. We then analyzed the role of simulated HIO_3_ and higher iodine oxides in the particle growth rates using a box model constrained by laboratory experiments. Furthermore, the influence of atmospheric O_3_ levels on HIO_3_ production was explored, along with the corresponding response in particle growth rates. These provide new insights into the linkage between IO radicals and further iodine-mediated nucleation and particle growth.

## 2. Methods

### 2.1 Reaction setup

**Fig. 1:**
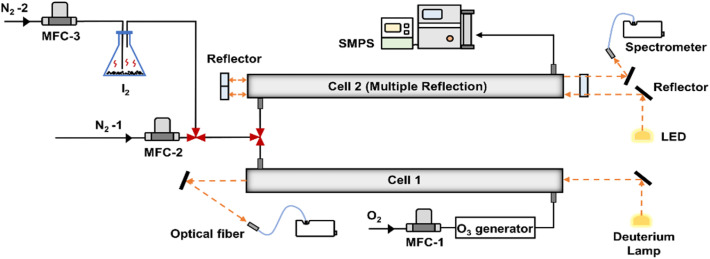
Schematic of experimental setup, with dual-optical cell DOAS design for measurement of IO generation.

The reactions and measurements were performed in two flow cells, as shown in Fig. 1. O_3_ produced from irradiation of oxygen (99.999%) under a UV lamp, was first flowed into Cell 1 and subsequently directed into Cell 2. Iodine crystals (≥99.8%) were placed in a transparent flask and their vapors were transported into Cell 2 using nitrogen (99.999%) as the carrier gas. The iodine abundance in Cell 2 can be adjusted by changing the flow rate of nitrogen gas or the weight of iodine crystals. In Cell 2, I_2_ was photolyzed under the lamp irradiation and rapidly converted to IO in the presence of O_3_ (R1 and R2), leading to iodine particle formation in the subsequent flow.

R1 I + *hv* → I + I

R2 I + O_3_ → IO + O_2_

The exhaust from Cell 2 was sampled using a Scanning Mobility Particle Sizer (SMPS, TSI) for measurement of the particle number size distribution. Detailed descriptions of the instrumentation are provided in the Text S1 of SI.

To increase the detection limit and measure IO at the pptv level, multiple reflectors were added at both ends of Cell 2 based on the white cell design principle.^47^ This configuration enabled the light beam to undergo multiple reflections within the cell, resulting in an effective optical path length of 20 m. The multiple-reflection configuration is shown in Fig. S1.

According to the different I_2_ levels entering Cell 2, the experiments were divided into three groups (E1, E2 and E3, see Table S1). Under otherwise identical conditions, varying the initial I_2_ concentration yielded different IO levels, allowing us to further investigate the role of IO in new particle formation and growth. Throughout the experiments, the flow rate of mass flow controller-1 (MFC-1) was maintained at 3 L min^−1^, equal to the sum of MFC-2 and MFC-3. In *E*_*m*−0_ (*m* = 1, 2, 3) experiments, two cells were cleaned by purified nitrogen gas. *E*_*m*−1_ experiments were carried out in the presence of I_2_ and purified oxygen gas with the O_3_ generator turned off. During *E*_*m*−*n*_ (*n* = 2, 3, 4, 5, 6) experiments, O_3_ was produced and injected into Cell 1 with the generator switched on. Meanwhile, the O_3_ mixing ratio was controlled by adjusting the irradiation area of the UV lamp. By keeping the sum of MFC-2 and MFC-3 flow rates constant, the I_2_ vapor concentration in Cell 2 remained stable at 1.33 ± 0.02, 0.74 ± 0.04 and 6.80 ± 0.26 ppmv, respectively. All experiments were conducted at a relatively stable temperature (*T*, 17.7 ± 0.6 °C) and relative humidity (RH, 5.5 ± 0.5%), with negligible variations across different experimental groups.

### 2.2 Retrieval of I_2_, O_3_ and IO concentration

Within the concept of dual-optical cell DOAS system, the concentrations of I_2_, O_3_, and IO were retrieved by DOAS technique. Spectral analysis was performed according to the Beer–Lambert law,^48^ using the DOASIS software developed by the Institute of Environmental Physics in Heidelberg University, Germany.^49^ IO was fitted in the spectral range of 426.3–447.9 nm using its absorption cross section,^50^ as well as reference of NO_2_ (ref. 51) and H_2_O (from The HITRAN Database). I_2_ was retrieved in the spectral interval of 532.4–547.3 nm and cross sections of I_2_,^52^ references of OIO^50^ and H_2_O were included. O_3_ (ref. 53) was evaluated in the range of 256.2–273.9 nm with no interference. The examples of spectral fitting for IO, I_2_ and O_3_ are presented in Fig. S2, and detailed fitting configurations and detection limits are summarized in Table S2. The effect of particle nucleation and growth on DOAS measurements has been described in the Text S2 and Fig. S3.

### 2.3 Box modelling

A box model is a zero-dimensional chemical model that simulates the temporal evolution of chemical species within a well-mixed box. It focuses on the production and loss of species *via* gas-phase and heterogeneous reactions under experimental or atmospheric conditions. The Framework for zero-Dimensional Atmospheric Modeling (F0AM),^54^ incorporating chemical kinetics from the Master Chemical Mechanism v3.3.1 (MCM), has been updated with state-of-the-art iodine chemistry (see Table S3). The modeling mechanisms and calculations are detailed in Text S3. The simulations were constrained by measured data, including *T*, RH, pressure, photolysis frequencies and mixing ratios of I_2_, O_3_ and IO. The photolysis rates of iodine-containing species were calculated based on the actinic flux, absorption cross section, and quantum yield. The cross section and quantum yield were taken from values recommended in the literature.^55^ The actinic flux of the LED lamp used in the experiment was measured in the laboratory and applied in the model simulations. Detailed photolysis rate calculation and values can be found in Text S3 and Table S4. Besides, additional heterogeneous chemistry (see Table S5) and dilution loss were considered in the model simulations.

## 3. Results and discussion

### 3.1 IO and particle generation

Experiments were performed under different conditions summarized in Table S1, with the corresponding temporal evolution of the I_2_, O_3_ and IO presented in Fig. S4. Clear IO generation was detected when both I_2_ and O_3_ were introduced simultaneously. Across all experiments (*E*_*m*_), IO production increased with rising O_3_ mixing ratios at near-constant I_2_ levels, resulting in measured IO values ranging from 0.24 to 0.90 ppbv (see Table S1 for precise gas-phase values). Despite the broad range of O_3_ input levels (0.05–29.47 ppmv), the highest IO yield (0.90 ppbv) was observed in E3 group with high iodine level, suggesting that elevated I_2_ mixing ratios enhance IO production. In E2 group with low iodine level, minimal increase in IO yield with rising O_3_ implies that the system is O_3_-saturated, wherein iodine availability becomes the rate-determining factor.

**Fig. 2:**
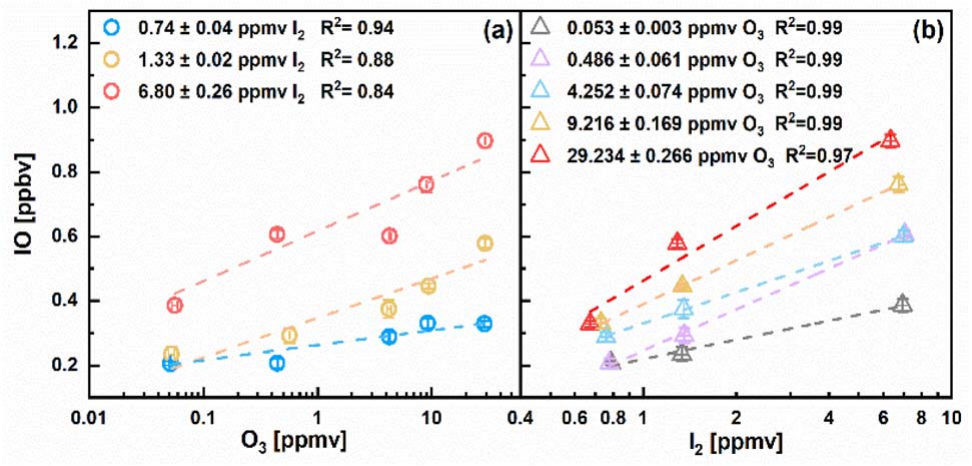
IO levels as a function of (a) O_3_ and (b) I_2_ mixing ratios across different experiments. Varying I_2_ and O_3_ levels are color coded. *T* and RH were maintained at 17.7 ± 0.6 °C and 5.5 ± 0.5%, respectively.

Dependence of IO production on different precursors (O_3_ and I_2_) concentrations is demonstrated in Fig. 2. For three given I_2_ levels, the IO mixing ratios as a function of O_3_ values exhibited correlation coefficients of *R*^2^ = 0.94, 0.88 and 0.84, respectively. As previously described, the slope of log_10_[O_3_] *versus* log_10_[IO] increased with higher I_2_ levels, indicating enhanced IO production efficiency at higher iodine availability. A similar trend can be observed in Fig. 2b, where IO production also responded strongly to changes in I_2_ at certain O_3_ levels. IO generation is influenced by both the formation rate of I atom and O_3_ concentration. A previous modeling study suggested that relatively low I_2_ concentrations observed in ambient air can still accelerate O_3_ depletion while simultaneously generating IO *via* reactions R1 and R2.^56^ At low I_2_ concentrations, IO formation is likely limited by the availability of iodine atoms.

**Fig. 3:**
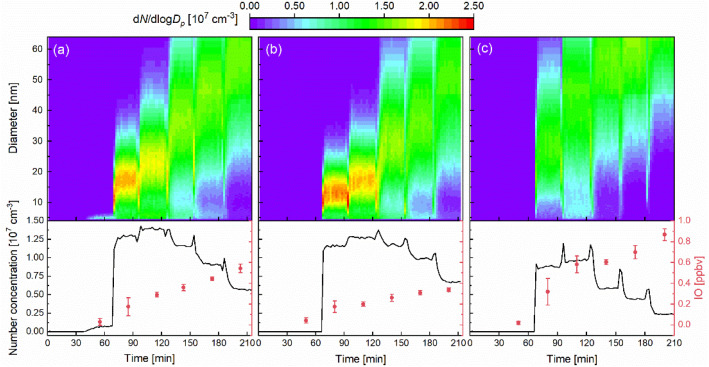
Temporal evolution of the particle number size distribution (top, color map), total particle number concentration (bottom, black lines) and IO mixing ratio (bottom, red dots) under different concentrations of I_2_ and O_3_ for (a) E1, (b) E2 and (c) E3. The color at each (time, diameter) pixel indicates the particle number concentration at that specific diameter and time, expressed as d*N*/d log *D*_p_ (cm^−3^). The spikes in the number concentrations were caused by disturbances resulting from the increases in O_3_ mixing ratio at each experimental step. Error bars represent ±1 standard deviation of IO values. All experiments were conducted under constant, continuous flow conditions with relatively stable of *T* (17.7 ± 0.6 °C) and RH (5.5 ± 0.5%). The specific I_2_ and O_3_ levels for each experiment are provided in Table S1.

Particle formation was consistently observed in all *E*_*m*−*n*_ experiments with simultaneous I_2_ and O_3_ introduction. Fig. 3 illustrates the temporal evolutions of particle number size distribution alongside corresponding IO mixing ratio. Following IO detection, a distinct new particle formation burst occurred, with peak number concentrations reaching 2.44 × 10^6^–1.42 × 10^7^ cm^−3^, corresponding to IO values of 0.21–0.90 ppbv. In previous laboratory studies of CH_2_I_2_/O_3_ photolysis system,^39,57^ maximum number concentration of 8.2 × 10^6^ cm^−3^ was observed by Jimenez *et al.* under conditions of 50 ppbv CH_2_I_2_ and 500 ppbv O_3_, and Wei *et al.* reported a mean number concentration about 4.5 × 10^7^ cm^−3^ at 1.3 ppmv CH_2_I_2_ and 0.5 ppmv O_3_. These values were comparable to this study. Similarly, under ambient light and 300 ppbv O_3_, the particles formed by iodine released from laminaria can reach 10^7^ cm^−3^.^58^ In chamber, laminaria exposed to radiation and initial 24 ppbv O_3_ can also produce particle with a peak number concentration of 6.8 × 10^7^ cm^−3^.^59^ However, in field observations conducted at Roscoff (maximum IO level ∼30 pptv), Mweenish Bay (peak IO level ∼35 pptv), and Helsinki (maximum high iodic acid concentration ∼3.2 × 10^7^ mol cm^−3^),^38,60,61^ the concentrations of iodine particles ranged from 10^4^ to 10^5^ cm^−3^, which were significantly lower than our experimental results. By comparing the correlation between particle number concentrations and IO values, this discrepancy can be attributed to the elevated IO levels under laboratory conditions.

In *E*_*m*−1_ experiments, when only I_2_ and oxygen were introduced, no NPF were observed, confirming that subsequent particle formation was not caused by the direct condensation of I_2_ vapor. Additionally, it is worth noting that in the E1-1 experiment, although I_2_ was introduced without O_3_, NPF with low concentrations (∼7.74 × 10^5^ cm^−3^) was detected due to a tiny amount of O_3_ generation (<detection limit) from O_2_ photolysis by the deuterium lamp's emission (*λ* < 242 nm),^62^ which in turn led to further IO and iodine particle formation. To avoid this situation, the deuterium lamp was covered with a baffle in E2-1 and E3-1 experiments, resulting in no particle formation. Besides, particle number concentrations and size distribution characteristics were obviously different with the variations in I_2_ and O_3_ mixing ratios, *i.e.*, generated IO levels. At lower IO concentrations, the particles were dominated by smaller sizes (<25 nm), indicating limited condensable vapor availability. As IO concentrations increased, there was a notable enhancement in particle size distribution toward larger diameters. The observed role of IO in particle behavior will be discussed in the following section.

### 3.2 Particle formation under different IO mixing ratios

**Fig. 4:**
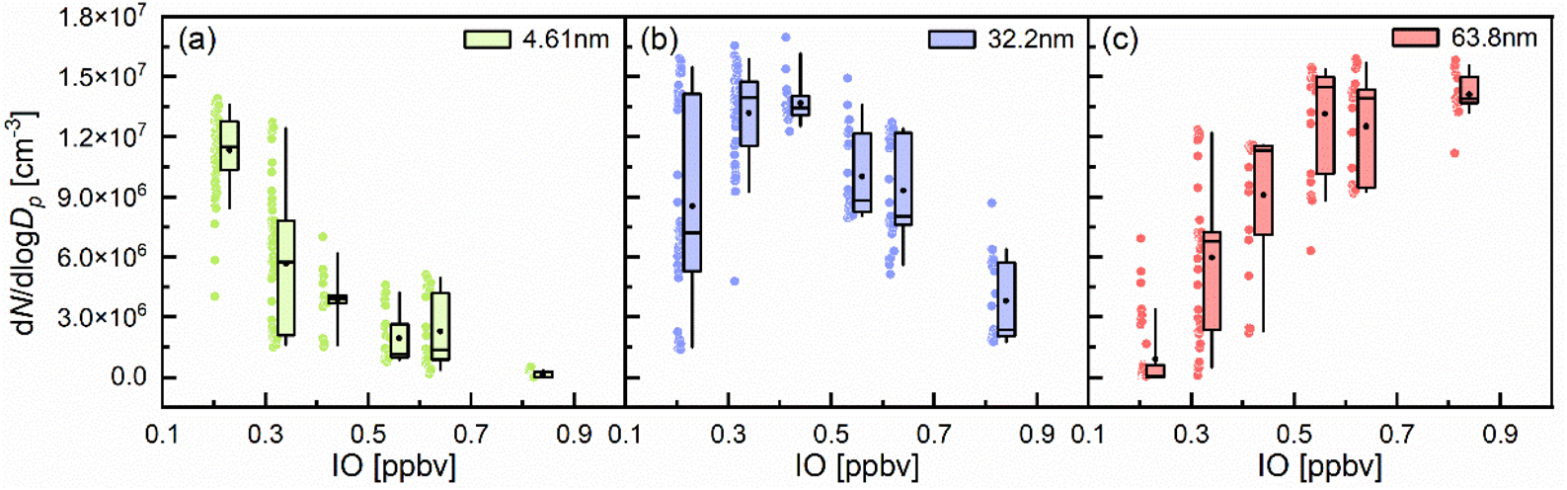
Particle number concentrations of three representative diameters (min, median and max) in varying IO mixing ratio bins. (a) 4.61 nm, (b) 32.2 nm and (c) 63.8 nm are shown in different colors. Data from all experiments (E1–E3) are aggregated into 0.1 ppbv IO bins; concentrations below 0.3 ppbv and above 0.7 ppbv are grouped into single bins. The bottom and top edges of the box indicate the first and third quartiles. The whiskers indicate the 5th and 95th percentiles. The internal lines and black dots represent median and mean values, respectively.

To facilitate the analysis, three representative particle sizes within detection range (minimum, median and maximum) were selected, namely 4.61, 32.2, and 63.8 nm. These correspond to nucleation mode (<25 nm) and Aitken mode (25–100 nm).^63^ IO levels were binned into 0.1 ppbv intervals, while concentrations below 0.3 ppbv and above 0.7 ppbv were aggregated into single bins, respectively. Notably, particle numbers at three representative particle sizes responded differently to increasing IO concentrations (Fig. 4). Concentrations of nucleation mode particles (4.61 nm) decreased progressively with higher IO, likely due to enhanced coagulation or condensational growth beyond this size range. For Aitken mode particles at 32.2 nm, concentrations peaked at 0.4–0.5 ppbv IO before declining, suggesting an optimal IO range for growth. For another Aitken mode particles at 63.8 nm, sustained growth with increasing IO was observed, with concentrations peaking above 0.7 ppbv (Fig. 4c), indicating continuous vapor condensation and particle aging. The inverse relationship between the smallest and largest particle sizes implies an IO-mediated shift of size distributions toward larger diameters *via* physicochemical processes. Furthermore, this trend suggests that IO may reflect the availability of iodine oxides and related condensable species. It can to some extent serve as an indicator of the gas-to-particle conversion processes, including particle nucleation and growth. Consistent with this, some field observations performed in coastal MBL have also reported positive correlations between IO levels and particle number concentrations in 2.5–10 nm size bins, and high IO levels were associated with a shift of the distribution toward larger sizes.^37,38^ However, unlike field settings where the influence of competing vapors (*e.g.*, sulfuric acid, organics) and meteorological factors may obscure the role of iodine chemistry, the controlled laboratory conditions of this study enable direct attribution of particle growth behavior to iodine-driven processes.

Fig. S5 displays the correlations between observed O_3_ and IO mixing ratios with mode diameter. The mode diameter depends on O_3_ levels under all three experimental conditions (E1–E3), with *R*^2^ values of 0.97, 0.96 and 0.92, respectively. Similarly, IO concentrations have good correlations with mode diameter, with *R*^2^ of 0.94, 0.87 and 0.88, respectively. Despite strong correlations between O_3_/IO concentrations and mode diameter, there are divergent trends in E1–E3, reflecting the fact that O_3_ and IO are not the ultimate precursors driving particle nucleation and growth. Prior studies^41,42^ have speculated that OIO and I_2_O_*y*_ (*y* ≥ 2) or higher iodine oxides are the key drivers of iodine particle formation and growth. However, other works^15,18^ have implicated HIO_3_ as the critical nucleating species, formed *via*:

R3 I_2_O_2_ + O_3_ → IOIO_4_

R4 IOIO_4_ + H_2_O → HIO_3_ + HOI + O_2_

To unify experimental observations, we assumed the sum of rates for IO reactions forming OIO and I_2_O_2_ as a proxy for the gas-to-particle conversion rate:

R5 IO + IO → OIO + I

R6 IO + IO → I_2_O_2_

R7 IO + O_3_ → OIO + O_2_

**Fig. 5:**
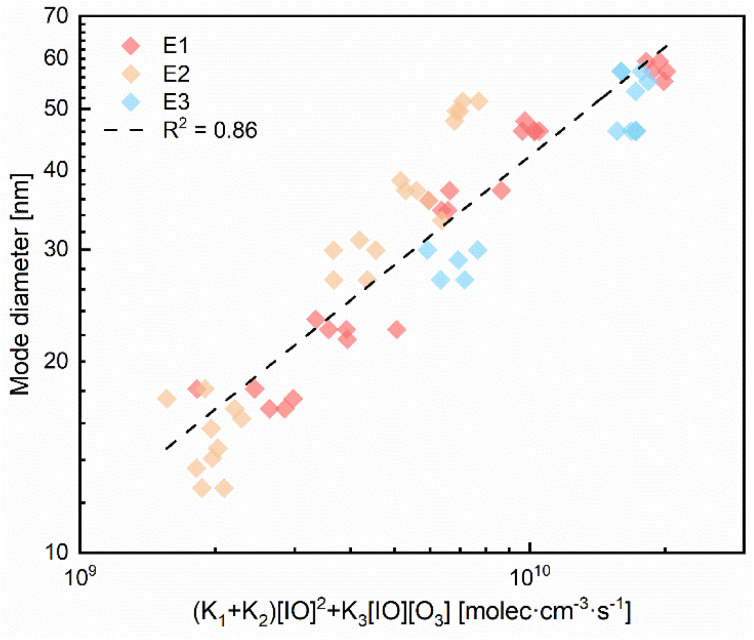
Measured mode diameter (the particle diameter at the peak of the number size distribution) as a function of the gas-to-particle conversion rate, estimated as rate = (*k*_1_ + *k*_2_) [IO]^2^ + *k*_3_[IO][O_3_], where *k*_1_, *k*_2_, and *k*_3_ are the rate constants for the IO self-reaction (forming OIO and I_2_O_2_) and the IO + O_3_ reaction, respectively. The three experimental groups (E1–E3) are distinguished by color.

1 Rate_gas-to-particle_ = (*K*_1_ + *K*_2_)[IO]^2^ + *K*_3_[IO][O_3_]

where *K*_1_ (4.0 × 10^−11^ cm^3^ per molecule per s, 291 K), *K*_2_ (6.1 × 10^−11^ cm^3^ per molecule per s, 291 K) and *K*_3_ (3.6 × 10^−16^ cm^3^ per molecule per s, 298 K) represent the rate constants for reactions of (R5–R7), respectively. Fig. 5 demonstrates the rate_gas-to-particle_ exhibits a good consistent trend with the mode diameter within E1–E3, reaching an *R*^2^ of 0.86. The result indicates that the rates of OIO and I_2_O_2_ formation follow a similar pattern to the particle growth dynamics. IO itself does not cause nucleation but modulates particle growth through subsequent reactions. Therefore, these findings underscore the importance of IO not only as a marker of reactive iodine chemistry but also as a trigger of new particle formation and growth pathways.

### 3.3 Effects of HIO_3_ and iodine oxides on particle growth

To further evaluate the roles of HIO_3_ and higher iodine oxides in particle growth, we conducted box model (F0AM) simulations constrained by laboratory measurements (*T*, RH, pressure, photolysis rates, O_3_ and I_2_ mixing ratios). To validate the accuracy of the model, simulated IO mixing ratios were compared with laboratory measurements. Due to the poor agreement between modeled and observed IO concentrations at high O_3_ levels, the reaction pathways between I_2_ and O_3_ were adjusted (see in Text S3). Fig. S6 shows the comparison between the simulated and measured IO concentrations before and after modifying the gas-phase iodine chemistry mechanism. After adjusting the branching ratio, the agreement improved significantly, with correlation coefficients (*R*) of 0.98, 0.90, and 0.93, and slope errors < 9%, 15%, and 23% for E1, E2 and E3, respectively (Fig. S7). The modified pathway shifts the branching toward OIO production and improves the model's ability to accurately reproduce IO concentrations at varying O_3_ levels. A summary of iodine chemical mechanism involved in the experiment is shown in Fig. S8. And a section on the scaling analysis of gaseous iodine chemical processes is discussed in Text S4, suggesting that our results can provide some mechanistic insights into the formation and growth of new particles.

**Fig. 6:**
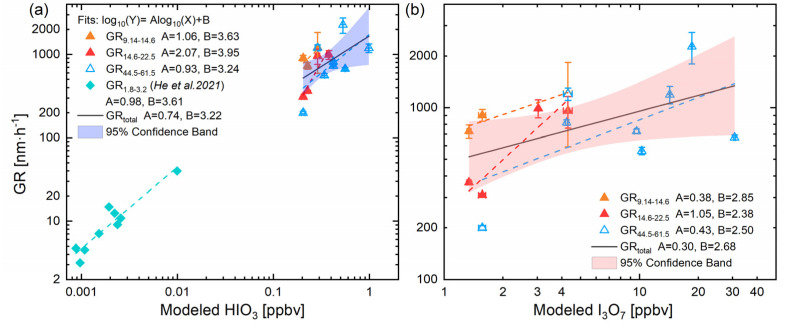
Growth rates of particles *versus* simulated (a) HIO_3_ and (b) I_3_O_7_ mixing ratios. The orange, red and hollow triangles represent growth rates for diameters of 9.14–14.6 nm, 14.6–22.5 nm and 44.5–61.5 nm with error bars of ±1 standard deviation, respectively. The diamonds in (a) indicate the previous measurement of HIO_3_ and growth rate in 1.8–3.2 nm. The fitting curves (solid lines) and 95% confidence bands (shaded regions) are presented for the combined data in each panel. The simulation parameters are set according to the experimental conditions in E1–E3 (Table S1).

Based on the measured particle number size distribution, particle growth rates (GR) were calculated for three size bins: 9.14–14.6 nm, 14.6–22.5 nm, and 44.5–61.5 nm, using the 50% appearance time method,^64,65^ as described in Text S5. Although HIO_3_ and I_3_O_7_ cannot be measured directly, we simulated them using the observation-constrained box model. Here, I_3_O_7_ serves as an indicator of higher iodine oxides. Fig. 6a shows the dependence of the concentrations of HIO_3_ on the growth rates in three size bins. For the 9.14–14.6 nm bin, GR increased from 904 to 1213 nm h^−1^ as HIO_3_ rose from 0.20 to 0.29 ppbv, following a fitted relationship of GR_9.14–14.6_ (subscripts denote the particle diameter range in nm) = 10^3.63^ × [HIO_3_]^1.06^ nm h^−1^. GR_14.6–22.5_ (310–991 nm h^−1^) followed a power law of 10^3.95^ × [HIO_3_]^2.07^ nm h^−1^ as HIO_3_ increased from 0.20 to 0.38 ppbv. For GR_44.5-61.5_ (200–2271 nm h^−1^), the fitting formula was 10^3.24^ × [HIO_3_]^0.93^ nm h^−1^, with HIO_3_ increasing from 0.20 to 0.98 ppbv. An exponent close to unity indicates that GR is approximately proportional to the available HIO_3_ concentration. For 9.14–14.6 nm, the Kelvin effect may be diminished due to high HIO_3_ levels. GR becomes limited primarily by the collision processes with negligible evaporation. For 44.5–61.5 nm, the Kelvin barrier falls with increasing size, and GR is governed by the collision rate which yields a linear dependence on vapor concentration. In addition, an exponent larger than unity for the 14.6–22.5 nm range indicates a superlinear response, which may arise from the dehydration or polymerization of two HIO_3_ molecules, or synergistic condensation of HIO_3_ and higher iodine oxides. Direct experimental evidence has established that both HIO_3_ and I_*x*_O_*y*_ participate in iodine-driven particle formation and can co-aggregate to form clusters.^20^ A study has reported that particle-phase accretion reactions can enhance GR beyond those predicted by partitioning alone, particularly for particles larger than 20 nm.^66^ And another study has pointed out that cluster–cluster collisions can significantly enhance GR at high vapor concentrations, demonstrated by cluster population simulations.^67^ HIO_3_-containing clusters formation, improved stabilization of clusters or rapid collision rate may be the reason for the exponent increase. It is noted that the absolute GR reported here are significantly higher than those observed in the ambient atmosphere. This is primarily due to the elevated I_2_ and O_3_ concentrations required to generate measurable particle formation and growth within the short residence time. The purpose of these experiments is not to replicate ambient GR values directly, but to elucidate the mechanistic dependence of particle growth on condensable iodine vapors under controlled conditions. He *et al.*^18^ reported that the GR between 1.8 and 3.2 nm at 10 °C was observed due to ambient HIO_3_ levels in the CLOUD experiment, which was fitted as 10^3.61^ × [HIO_3_]^0.98^ nm h^−1^. A similar trend was observed between CLOUD measurements and our study (GR_9.14–14.6_ and GR_44.5–61.5_), showing near-linear responses despite differences in size bins, HIO_3_ concentrations, and environmental conditions (T and RH). Collectively, these findings suggest that HIO_3_ is closely associated with iodine particle growth across the studied size bins.

By contrast, the lower slope (*A*) in Fig. 6b suggests that the dependence of GR on I_3_O_7_ is weaker than that on HIO_3_, except for the particle size range of 14.6–22.5 nm. A laboratory study using flow tube suggests that I_2_O_*y*_ predominantly contribute to iodine particle formation under extremely dry conditions (RH< 1%).^20^ And CLOUD chamber experiments found that when RH exceeds 2%, iodine oxoacid mechanism (HIO_3_ × HIO_2_) can control the rate of iodine particle formation.^68^ One possible explanation for this discrepancy is the role of photolysis. Photolysis of key intermediates, such as IO, OIO, and I_2_O_*y*_, reduces the steady-state concentrations of higher iodine oxide clusters, thereby limiting their ability to participate in further clustering or condensation onto growing particles. By contrast, HIO_3_ is relatively more stable under light, allowing it to accumulate and act as a more effective condensable vapor. Additionally, HIO_3_ can undergo heterogeneous reactions to form IO_3_^−^ ions, which exhibit low volatility and high hydrophilicity.^69,70^ This stability facilitates its role in particle growth, particularly *via* condensation or hydrolysis onto pre-existing clusters or freshly nucleated particles. Therefore, these results suggest that HIO_3_ may be a key species governing both iodine particle nucleation and growth, even under relatively dry atmospheric conditions. These findings may be relevant to localized iodine-rich environments under dry conditions, such as salt lake, mid-latitude inland areas or polar source regions. However, the present laboratory results should be interpreted as mechanistic insight rather than a quantitative representation of the ambient atmosphere. Because HIO_3_ and higher iodine oxides were not directly measured, this interpretation should be regarded as model-dependent and subject to uncertainty. In addition, alternative pathways involving higher iodine oxides, mixed iodine clusters, or cooperative mechanisms cannot be excluded. The role of higher iodine oxides may become more prominent when particle formation shifts from nucleation mode towards small Aitken mode, which deserves to be further investigated through experimental and modeling approaches. Due to the short residence time, using high concentration reactants in laboratory flow cells is a common approach for studying halogen chemistry.^20,42,57^ While laboratory studies on iodine particle nucleation and growth are not conducted under atmospheric conditions, where high concentrations of iodine oxides may drive particle formation and growth *via* dipole–dipole enhanced second-order chemistry. This does not preclude the possibility that HIO_3_-driven mechanisms also govern iodine particle nucleation and growth. In fact, both pathways may compete under certain environmental conditions.^20^ The strong correlations between IO levels and particle size shifts, and between modeled HIO_3_ and GR, reveal a mechanistic dependency that may remain consistent across a broad concentration range.

A concentration matrix of IO and O_3_ was designed for the simulations of HIO_3_ and I_3_O_7_ (see in Text S6), and the results are shown in Fig. S10a and S10b. The concentrations of HIO_3_ are not linear with O_3_ and IO mixing ratios. The low levels of either O_3_ or IO are unfavorable for HIO_3_ formation. Simulated I_3_O_7_ concentrations remain generally low at IO levels below 0.2 ppbv, but increase when IO exceeds 0.5 ppbv, especially with increasing O_3_ concentrations. Fig. S10c presents the laboratory-observed GR under the same O_3_ and IO gradients. It can be seen that GR_9.14–14.6_ increases with rising IO concentrations (0.21–0.39 ppbv) at comparable O_3_ levels, corresponding to increased HIO_3_ and I_3_O_7_ values. As the IO (0.21–0.39 ppbv) and O_3_ (52–445 ppbv) levels increase individually, the GR_14.6–22.5_ exhibits a rising trend, again corresponding to higher HIO_3_ and I_3_O_7_ concentrations. The GR_44.5–61.5_ reaches a maximum of 2271 nm h^−1^ when higher IO (0.45 ppbv) and O_3_ (9396 ppbv) concentrations present. Both IO and O_3_ critically regulate the formation of HIO_3_ and higher iodine oxides, which in turn control size-dependent particle growth. The close correspondence between the simulated condensable vapor and the observed GR highlights the joint influence of iodine radicals and oxidants when evaluating iodine-mediated nucleation and particle growth.

**Fig. 7:**
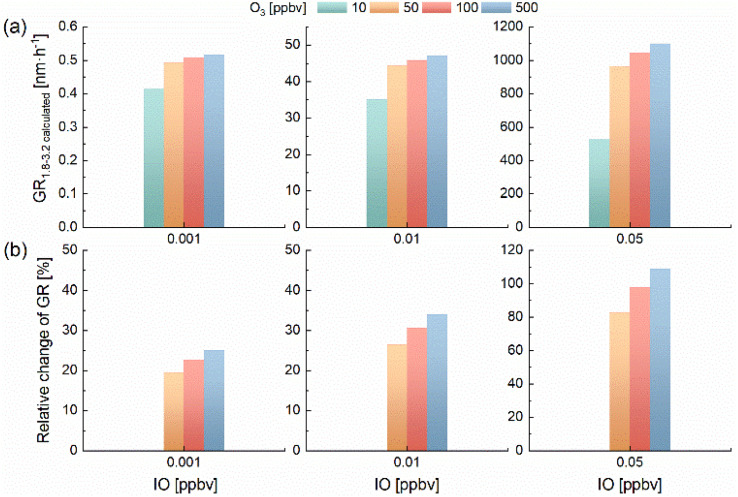
(a) Calculated GR_1.8–3.2_ and (b) relative change of GR_1.8–3.2_ under varying O_3_ and IO levels. GR_1.8–3.2_ calculated by fitting formula from CLOUD experiment, based on modeled HIO_3_ levels. The relative change ratio of calculated GR_1.8–3.2_ compared to the base case of 10 ppbv O_3_ at different IO levels.

To assess HIO_3_ and I_3_O_7_ production at ambient levels of O_3_ and IO, we analyzed simulations constrained to O_3_ below 500 ppbv and IO below 0.05 ppbv. Under these conditions, HIO_3_ increases with O_3_, most sharply between 10 and 50 ppbv (Fig. S11a), while I_3_O_7_ exhibits an even stronger response (Fig. S11b). Due to the relatively low modeled HIO_3_ concentrations (8.42 × 10^−5^–0.26 ppbv), the GR_1.8–3.2_ was calculated using the fitting formula derived from CLOUD experiment.^16^ In Fig. 7a, as O_3_ rises from 10 to 500 ppbv, the calculated GR_1.8–3.2_ also increases, ranging from 0.4–0.5, 35.2–47.2 and 526.9–1099.8 nm h^−1^ at IO concentrations of 0.001, 0.01 and 0.05 ppbv, respectively. Fig. 7b illustrates the relative changes in GR_1.8–3.2_ compared with the base scenario of 10 ppbv O_3_ at each IO level. When O_3_ increases from 10 to 50 ppbv, the relative change in GR_1.8–3.2_ reaches 20%, 26%, and 83% for the three IO scenarios, suggesting a possible role of O_3_ in promoting HIO_3_ formation. By contrast, changes in GR_1.8–3.2_ become less distinct when O_3_ concentration exceeds 50 ppbv, suggesting that variations in lower O_3_ concentration range (<50 ppbv) have a stronger influence on HIO_3_ formation. Analysis of the effect of O_3_ on HIO_3_ (in Text S7) shows that at low O_3_ levels, O_3_ promotes the formation of HIO_3_, however, at high concentrations, O_3_ primarily influences HIO_3_ indirectly by affecting the formation of IO. O_3_ variability may influence iodine chemistry, particularly the formation of HIO_3_ and higher iodine oxides by coupled processes (Fig. S8), although the magnitude of this influence in the ambient atmosphere remains to be quantified. The resulting increases in HIO_3_ and higher iodine oxides concentrations may, in turn, impact new particle formation and growth in polar, coastal and other iodine-rich environments. Previous researches have suggested that humidity may regulate the balance between iodine oxides and HIO_3_, with increased humidity favoring the conversion toward HIO_3_.^15,68^ However, the role of O_3_ in altering the relative abundance of HIO_3_ and I_*x*_O_*y*_ remains insufficiently understood and warrants further investigation. To accurately assess iodine-mediated particle formation and growth under diverse atmospheric conditions, it is essential to comprehend the O_3_-driven shifts in the proportions of iodine species.

## 4. Conclusions

This study was conducted using a newly designed dual-optical cell DOAS system and SMPS to investigate iodine particle formation and growth under controlled laboratory conditions (*T* = 17.6 °C, RH = 5.5%). The experimental results indicate that IO radical levels (0.21–0.9 ppbv) are positively correlated with both particle number concentrations (2.44 × 10^6^–1.42 × 10^7^ cm^−3^) and size distribution. IO radicals can promote the formation of intermediate species during subsequent physicochemical processes, thereby catalyzing new particle formation and driving particle growth. The total rate of IO conversion to OIO and I_2_O_2_ closely followed the variation in the particle mode diameter (*R*^2^ = 0.86), underscoring the key role of these oxide intermediates in the growth dynamics.

Combined laboratory measurements and box modeling, particle growth rates in three size bins (9.14–14.6 nm, 14.6–22.5 nm, and 44.5–61.5 nm) displayed power-law relationships with simulated HIO_3_ concentrations. Similar trends in GR_9.14–14.6_ and GR_44.5–61.5_ have been found in previous studies of CLOUD measurements. This indicates a possible mechanistic link between HIO_3_ and particle growth, although the interpretation remains model-dependent due to the lack of direct measurement of HIO_3_ and higher iodine oxides. Model sensitivity simulations further show that in the three IO concentration scenarios (0.001, 0.01 and 0.05 ppbv), elevated O_3_ concentrations from 10 to 50 ppbv resulted in increases in GR_1.8–3.2_ of 20%, 26%, and 83%, respectively. These results suggest that even at low atmospheric iodine levels, variations in O_3_ may influence iodine chemistry and new particle formation by coupled processes.

Our findings highlight the need to consider both iodine precursors and oxidant levels when assessing the contributions of iodine to particle formation and growth. Although the high concentrations of precursors in experiments are feasible for studying potential chemical mechanisms, direct extrapolation of these findings to environmental conditions requires further investigation under more representative of atmospheric conditions. Nevertheless, the integrated experimental setup and modeling framework established here provides a methodological foundation for future quantitative assessments of iodine-driven nucleation and its impacts on aerosol loading and air quality.

## Author contributions

Yuhao Yan designed and implemented the research and prepared the paper. Shanshan Wang and Bin Zhou guided and supervised the research. Zhiwen Jiang and Chuanqi Gu contributed to the conduct of the experiments. Shuyao Xiang contributed to the writing and editing of the paper. Alfonso Saiz-Lopez provided the constructive suggestions for this study. All authors participated in the discussion and interpretation of the data.

## Conflicts of interest

The authors declare no competing financial interest.

## Supplementary Material

RA-016-D6RA01191H-s001

## Data Availability

The data are available from the corresponding author upon reasonable request. Supplementary information (SI) is available. See DOI: https://doi.org/10.1039/d6ra01191h.
