# Supplementary material for: Iodine-mediated nucleation and particle growth: laboratory measurements of IO production and its implications
Source: RSC Adv. 2026 May 26;16(31):28243–53. doi: 10.1039/d6ra01191h (PMC13213581; doi:10.1039/d6ra01191h)
Supplement: RA-016-D6RA01191H-s001 [file RA-016-D6RA01191H-s001.pdf]

Supplementary Information (SI) for RSC Advances.
This journal is © The Royal Society of Chemistry 2026

Supporting Information for

Iodine-Mediated Nucleation and Particle Growth: Laboratory Measurements of IO Production and Its Implications

Yuhao Yan,[a] Shanshan Wang,*[ab] Zhiwen Jiang,[a] Chuanqi Gu,[a] Shuyao Xiang,[a] Alfonso Saiz-Lopez [c] and Bin Zhou*[abd]

[a] Shanghai Key Laboratory of Air Quality and Environmental Health, Department of Environmental Science and Engineering, Fudan University, Shanghai 200433, China.

[b] Institute of Eco-Chongming (IEC), Shanghai 202151, China.

[c] Department of Atmospheric Chemistry and Climate, Institute of Physical Chemistry Blas Cabrera, CSIC, Madrid 28006, Spain.

[d] Institute of Atmospheric Sciences, Fudan University, Shanghai 200433, China.

Corresponding author: Shanshan Wang (shanshanwang@fudan.edu.cn) and Bin Zhou (binzhou@fudan.edu.cn)

Text S1. Instrumentation

Cell 1 was made of Teflon coated quartz glass with length of 1.13 m and diameter of 5.0 cm. Cell 2 was 1.0 m long and 5.0 cm in diameter, and constructed from Teflon-coated stainless steel.

Gas-phase species were measured using the Differential Optical Absorption Spectroscopy (DOAS) technique, which is fundamentally based on the Beer-Lambert law.[1] O_3 was measured by a deuterium lamp (HAMAMATSU L6311-50) and a spectrometer (B&W TEK Inc. BRC741E-1024) in 1-minute time resolution. The Teledyne Princeton Instruments spectrometer (HRS 300, grating 600 grooves mm^{-1}) with 1340×400 pixels detector (PIX-400B) and 5500K LED lamp (OSRAM LZ1-10CW02-0055) were used to measure I_2 and IO spectra with 2-minute time resolution.

The particle number size distribution of particle, ranging from 2 nm to 150 nm, was measured using a Scanning Mobility Particle Sizer (SMPS, TSI 3936) with 2-minute time resolution and 97 channels, positioned at the exit of Cell 2.

Experiments were carried out at temperature (T) of 17.7 ± 0.6 °C and 5.5 ± 0.5% relative humidity (RH), which were detected by the sensor at the exit of Cell 2.

Text S2. Effect of nucleation and particle growth on DOAS measurement

To quantitatively evaluate the effect of nucleation and particle growth on DOAS retrievals, we used the root-mean-square (RMS) of the spectral fitting residual as an indicator. The RMS quantifies the remaining spectral structures that cannot be explained by the fitted trace-gas references and the broadband baseline. And RMS can be affected by factors such as system noise, Mie scattering, stray light, excessive gas concentration and unaccounted interfering absorbers.[2,3] If newly formed particles and particle growth cause significant extinction (e.g., Mie scattering or broadband absorption) during the experiment, the residual between the measured and fitted spectra will increase, resulting in a higher RMS and larger measurement errors.

We compared the RMS values of the fitted gas-phase species at different stages of the experiment, including non-nucleation, nucleation and particle growth, as shown in Figure S3. For I_2 retrieval, the RMS did not show any systematic increase with nucleation and particle growth. This indicates

that under our experimental conditions, nucleation and particle growth have a negligible effect on the I_2 value derived from DOAS. For IO retrieval, although the RMS values appeared to increase with particle growth, they remained below 3×10^{-4}. This increase is likely due to enhanced extinction caused by the rising IO concentrations rather than by particles. Even at the highest RMS, the corresponding detection limit for IO is 0.4 ppbv, which is lower than the measured IO concentrations (0.90±0.02 ppbv). Therefore, the interference remains within an acceptable range and can be considered negligible for IO as well.

It should also be noted that the RMS values for I_2 are much larger than those for IO. This is because the measured I_2 concentration is on the order of ppmv, while IO is on the order of pptv. The high I_2 level causes significant extinction, which in turn leads to a larger RMS value.

Text S3. Box model description

Gas-phase iodine reactions and reaction rate constant expressions (*k*) were mostly taken from recent studies (Table S3). The latest mechanisms of HIO_3 formation reported by Finkenzeller et al.[4] were incorporated into the model. Thermal decomposition reaction rate constant expressions are also listed in Table S3. Under the modeled conditions, I_2O_3 was predicted to be thermally stable.[5]
Additionally, the branching ratio for reaction of I_2 with O_3 was modified based on experimental results. Comparison of IO measurements with simulations revealed deviations at higher O_3 concentrations (Figure S6). Since the I_2 and O_3 reaction products were uncertain,[5,6] the simulation was improved by changing the branching ratio. Significant improvements were observed when the branching was changed from 1:1 to 7:1, with correlation coefficients (R) between observed and simulated values exceeding 0.9 and slope error below 23%. The modified branching ratio was applied in subsequent simulations.

$$I_2 + O_3 \rightarrow IO + OIO \qquad K = 3.5 \times 10^{-10} \times exp(-2050/T) \qquad \text{(SR1)}$$

$$I_2 + O_3 \rightarrow IO + I + O_2 \qquad K = 0.5 \times 10^{-10} \times exp(-2050/T) \qquad \text{(SR2)}$$

To quantify the photolysis rates of iodine-containing species, the actinic flux was measured in the

laboratory. Cross sections and quantum yields of iodine species were taken from recommended values in literature.[7] The cross sections of I_2O_2, I_2O_3 and I_2O_4 used in model were predicted in previous studies.[8] The specific calculation formula is as follows:

$$Photolysis\ rate\ (j) = \int \sigma(\lambda)\Phi(\lambda)F(\lambda)\ d\lambda \qquad \text{(S1)}$$

Where σ, Φ, and F are cross section, quantum yield and actinic flux, respectively. λ represents wavelength.

The heterogeneous uptake rates onto aerosol for each species are calculated using:[9]

$$k_{het} = 0.25 \times \gamma \times S_a \times c \qquad \text{(S2)}$$

Where γ is the reactive uptake coefficient, S_a is the aerosol volumetric surface area obtained by SMPS, and c is the mean molecular speed of gas molecule. The mean molecular speed of gas molecule is calculated as follows, where R is the molar gas constant, T is the temperature and M is the molar mass.

$$c = \sqrt{\frac{8RT}{\pi M}} \qquad \text{(S3)}$$

While dilution loss is considered in the model, which is estimated by Eq. (S4), where Q is the flow rate and V is Cell 2 volume. The dilution loss approximately is $9.7 \times 10^{-3}\ s^{-1}$, and is treated as a permanent loss for each trace gas.

$$k_{dilution} = \frac{Q}{V} \qquad \text{(S4)}$$

Text S4. Scaling analysis of gaseous iodine chemical processes

To examine the scaling behavior of gas-phase iodine chemistry, additional box-model simulations were conducted under more atmosphere-relevant I_2 and O_3 levels, including Case1 (I_2 = 50 pptv and O_3 = 1, 2, 5, 10, 25, and 50 ppbv) and Case2 (I_2 = 100 pptv and O_3 = 1, 2, 5, 10, 25, and 50 ppbv). Following the actual experimental procedure, simulations were performed with I_2 level held constant and O_3 concentration was varied sequentially at 30-minute intervals. The simulated IO responses show a trend consistent with the laboratory observations, with similar slopes but different

intercepts, as shown in Figure S9. This indicates that iodine oxides formation may follow the same underlying chemical principles across a broad concentration range, whereas the absolute IO abundance is strongly affected by the precursors. The difference in intercepts is expected because of variations in precursor concentrations between laboratory and atmospheric environment. Therefore, the gaseous iodine chemical processes observed in the laboratory should be interpreted as scaling-relevant, and results provide mechanistic insight into the formation and growth of new particles.

Text S5. Particle growth rate calculation

The particle growth rates were calculated by the 50% appearance time method, which was used in many laboratory experiments.[10-12] Specific calculation procedure was described in previous studies.[13,14] The reaching time of 50% maximum in each size-channel can be fitted individually by a four-parameter sigmoid function:[11]

$$S_{dp}(t) = \frac{a-b}{1+\left(\frac{t}{t_{app}}\right)^{d}} + b \qquad \text{(S5)}$$

where dp represents the diameter size, a and b are the background and plateau values of the sigmoid function respectively, d is a parameter for the steepness of the rising signal, t_{app} is the reaching time of 50% value between background and plateau.

Text S6. HIO_3 and I_3O_7 simulation under a concentration matrix of IO and O_3

Multiple model scenarios were designed to simulate HIO_3 and I_3O_7 formation based on the atmospheric and laboratory concentrations of gas-phase precursors. The IO concentrations were set to 0.001, 0.01, 0.05, 0.2, 0.3, 0.4, 0.6 and 1.0 ppbv, while O_3 concentrations were set to 10, 50, 100, 500, 1000, 5000, 10000, and 30000 ppbv. Simulations were performed with fixed O_3 and IO levels for 10 minutes, using environmental parameters consistent with the experimental conditions of 17 °C and 5% RH. The specific simulation results are shown in Figure S10.

Text S7. Sensitivity analysis of HIO_3 and IO response to changes in O_3 concentration

It is important to recognize that O_3 serves multiple functions in the iodine oxidation processes. O_3 can react with iodine atoms to form IO, thereby further affecting the concentration of I_2O_2, or it can react directly with I_2O_2 and then hydrolysis to HIO_3. Consequently, the observed relationship between O_3 and particle growth reflects the integrated effect of O_3 across these interconnected pathways. We used measured I_2, O_3 and IO levels together with model simulations to assess the effect of O_3 on the formation of iodine oxides, as shown in Figure S12. In each experimental group, the variation in O_3 concentration exerted a stronger influence on the production rate of IO (pIO) than on those of I_2O_2 (pI_2O_2) and HIO_3 ($pHIO_3$), while its effects on I_2O_2 and HIO_3 were of comparable magnitude. A sensitivity analysis was conducted on the response of HIO_3 and IO to changes in O_3 concentration. It was found that the $pHIO_3/pI_2O_2$ ratio remained relatively consistent across the three experimental groups and did not show significant variation with O_3, except for a certain degree of dependence on O_3 at lower concentrations. This behavior likely arises from the influence of I_2O_2 sinks under these conditions.[4] In the conversion of I_2O_2 to HIO_3, O_3 is not the rate-limiting step, while the formation of I_2O_2 is the determining factor. In contrast, pIO/pI· (production rate of iodine atom radical) ratio varied markedly with O_3 concentration, indicating that O_3 significantly affects IO formation. Given that I_2O_2 is produced via the self-reaction of IO, this implies that O_3 influences I_2O_2 levels primarily through its effect on IO formation, and thereby indirectly regulates HIO_3. Consequently, under the experimental conditions, the promotion of particle growth by O_3 is achieved by indirectly influencing HIO_3 through increased IO formation. Li et al. provided field evidence from nocturnal measurements showing that O_3 as an oxidant participates in multiphase processes and promotes the formation of HIO_3.[15] Long-term HIO_3 measurements in polluted urban environments has been reported that HIO_3 concentration exhibits a clear diurnal pattern peaking around noontime, closely tracking atmospheric solar radiation and O_3 levels.[16] And they demonstrated that HIO_3 enhances the survival probability of sub-3 nm particles by about 40%.

Text S8. Sensitivity analysis of I_2 + O_3 branching ratio

To assess the impact of the modified I_2 + O_3 branching ratio on the model results, additional simulations were performed using the original branching ratio and the modeled HIO_3 and I_3O_7 concentrations were compared with the measured GR before and after adjusting the branching ratio. As shown in Figure S13b, both HIO_3 and I_3O_7 still exhibit positive relationships with GR, indicating that the qualitative dependence of particle growth on iodine oxidation products is preserved. At the same time, the absolute magnitudes of the simulated HIO_3 and I_3O_7 concentrations, as well as the fitted intercepts, vary with the branching ratio assumption, implying that these quantities remain subject to model uncertainty. In particular, HIO_3 remains more strongly associated with GR than I_3O_7, suggesting that HIO_3 may be the more relevant contributor under the present dry laboratory conditions. However, the absolute modeled concentrations do change, which introduces uncertainty into the quantitative estimates of HIO_3 and I_3O_7. These species should be interpreted as sensitivity-dependent estimates constrained by the observed IO data, rather than as validated absolute values. The main conclusions of the study are based more on the robustness of the observed trends, not on the exact numerical values of the simulated intermediates.

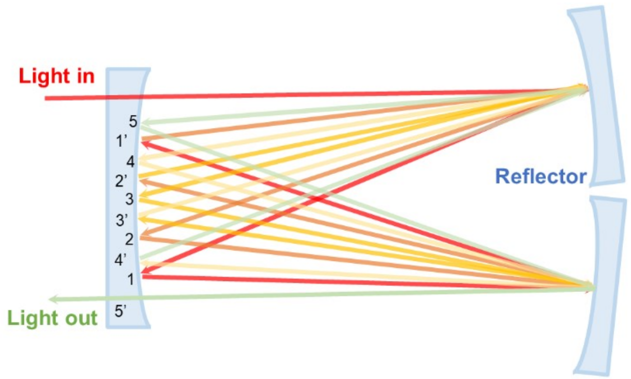

Figure S1. View of the multiple reflection cell for 20 passes. The focal points are numbered along the optical path (1, 2, 3, 4, 5 and 1', 2', 3', 4', 5').

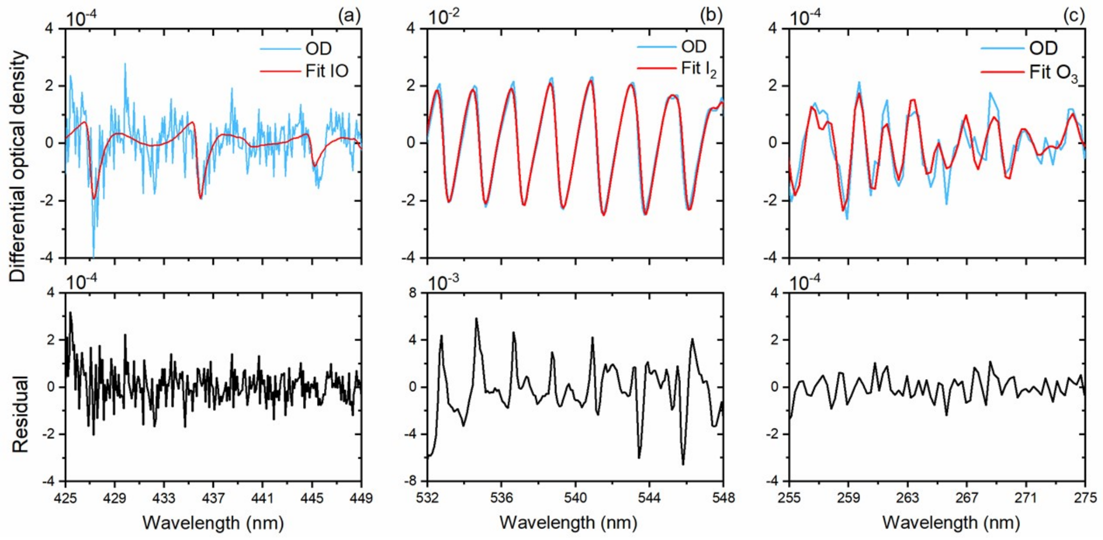

Figure S2. Examples of spectral fitting. (a) IO, (b) I_2 and (c) O_3 with differential optical density (OD, blue line), fitting result for each trace gas (red line) and residual structure (black line).

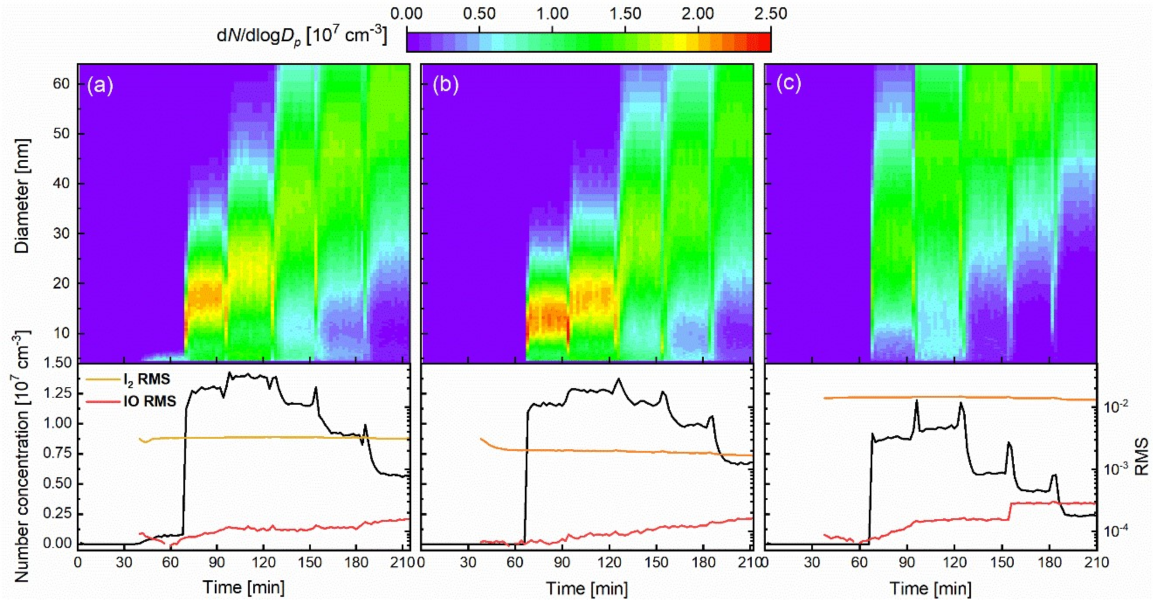

Figure S3. Temporal evolution of the particle number size distribution and RMS of I_2 (orange line) and IO (red line) for (a) E1, (b) E2 and (c) E3.

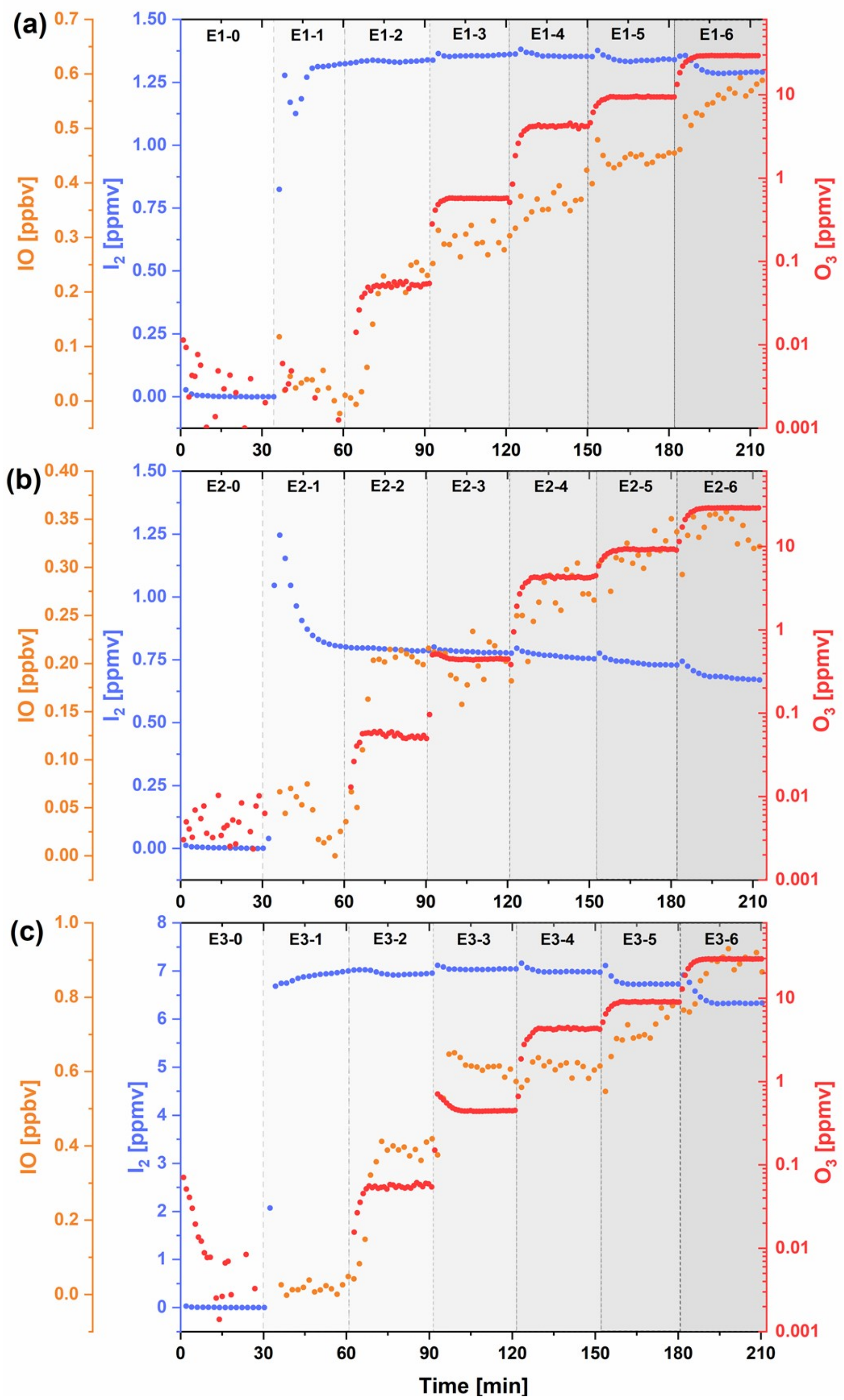

Figure S4. The time evolution of I_2, O_3 and IO mixing ratios throughout (a) E1, (b) E2 and (c) E3.

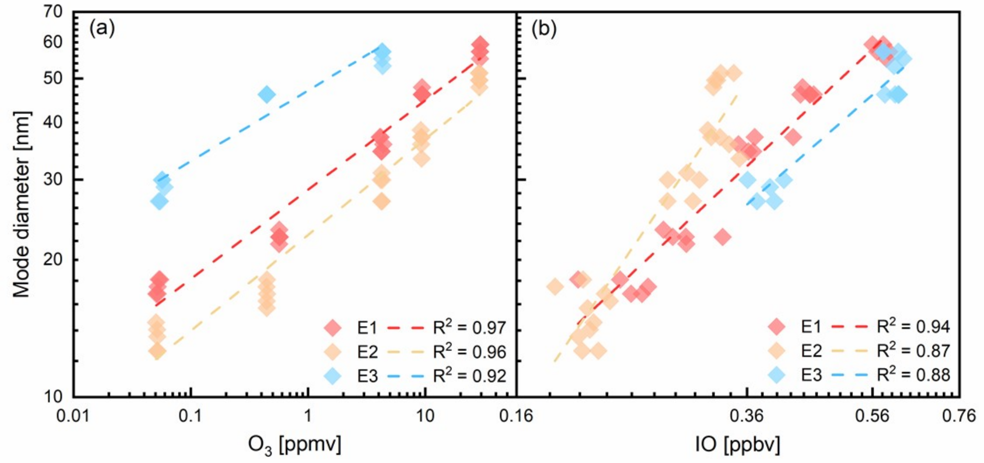

Figure S5. Correlation of observed (a) O_3 and (b) IO mixing ratios with mode diameter.

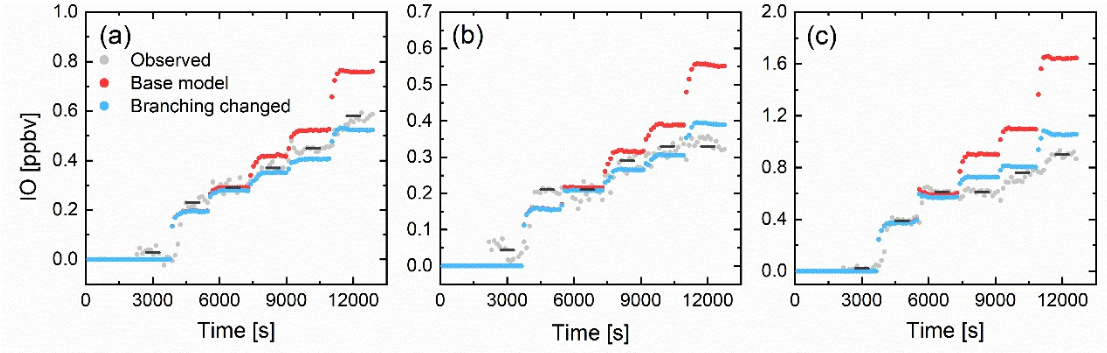

Figure S6. The temporal variations of IO values by observed and simulated in (a) E1, (b) E2 and (c) E3.

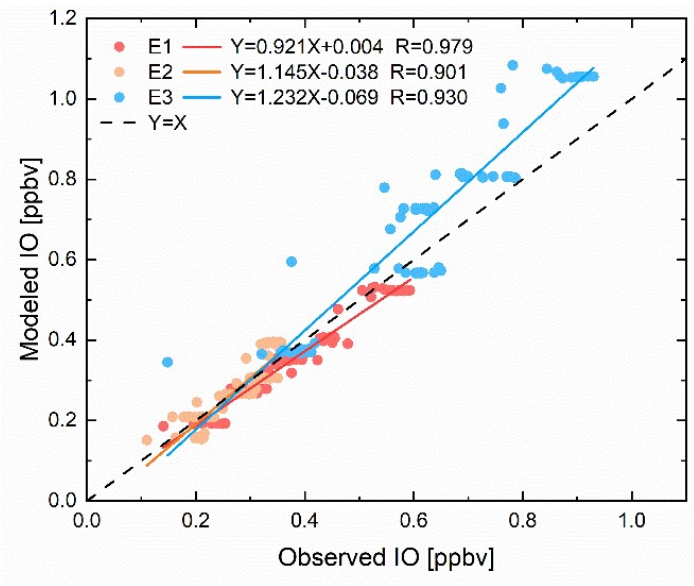

Figure S7. Correlation of IO mixing ratios by observed and simulated.

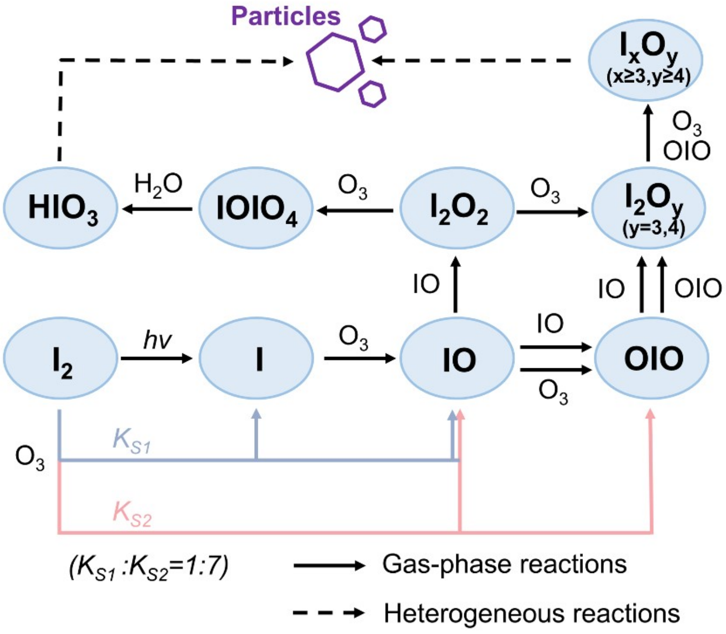

Figure S8. A summary of iodine chemical mechanism involved in the experiment.

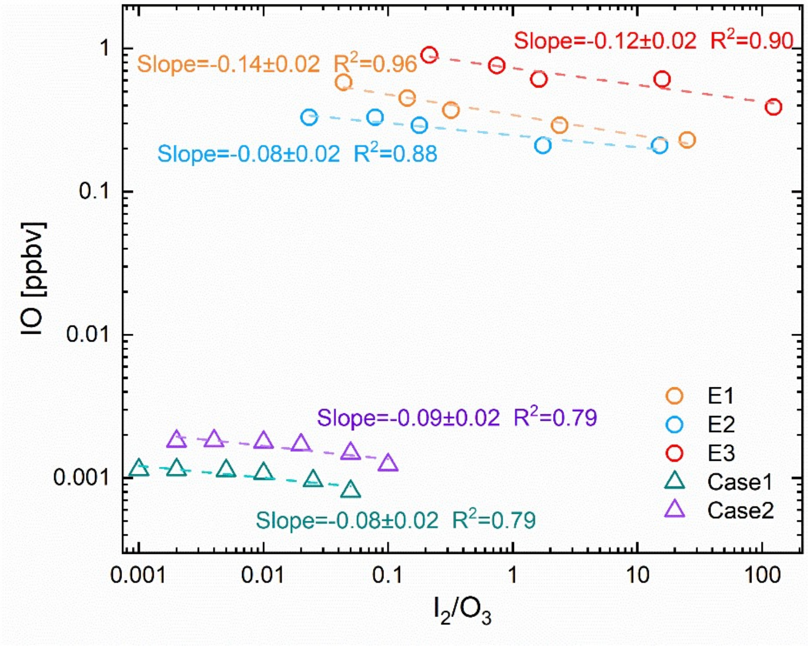

Figure S9. The response relationship between IO and I_2/O_3, including laboratory experiments (E1, E2 and E3) and simulation results (Case1 and Case2).

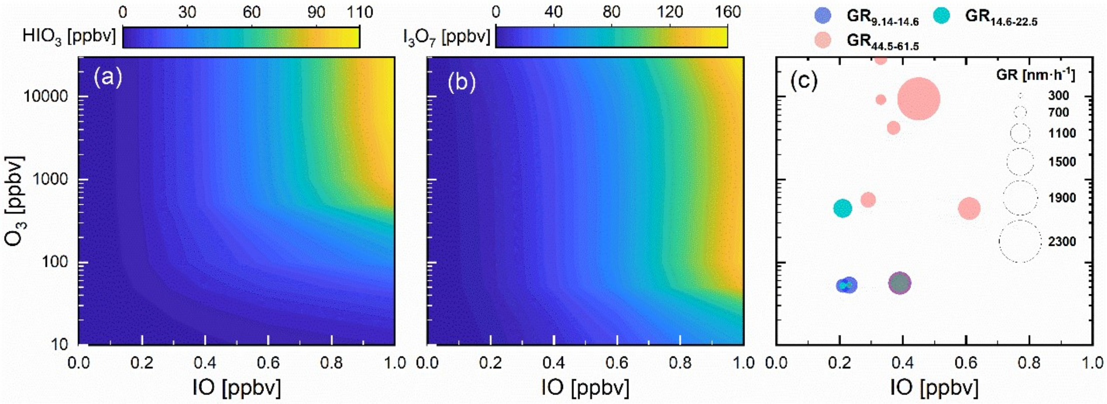

Figure S10. Simulated values of (a) HIO_3, (b) I_3O_7 based on O_3 (10-3×10^4 ppbv) and IO (0-1 ppbv) mixing ratios, and (c) particle growth rates in three size ranges (9.14-14.6 nm, 14.6-22.5 nm and 44.5-61.5 nm) under varying O_3 and IO mixing ratios in (a). The basic simulation parameters were set according to the experimental conditions in the flow tube, with a temperature of 17 °C and a relative humidity of 5%. The simulation was performed with fixed concentrations of O_3 and IO, and the simulation duration was 10 minutes.

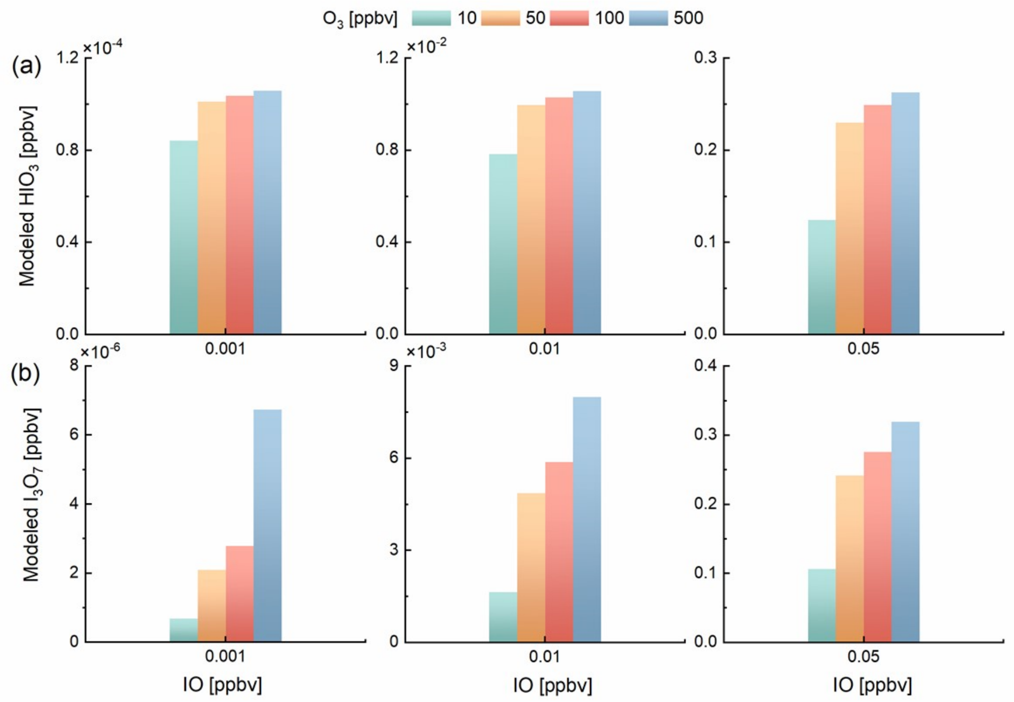

Figure S11. Simulated (a) HIO_3 and (b) I_3O_7 concentrations under different O_3 and IO levels. The basic simulation parameters were set according to the experimental conditions in the flow tube.

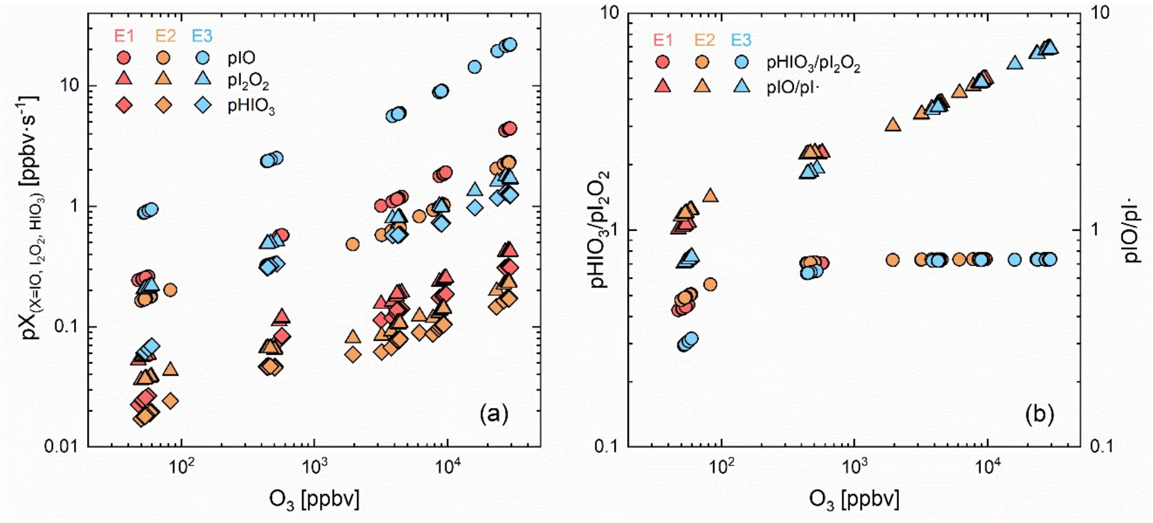

Figure S12. Variation in (a) production rates of X (X = IO, I_2O_2 and HIO_3), and (b) $pHIO_3/pI_2O_2$ and $pIO/pI\cdot$ with O_3 concentrations.

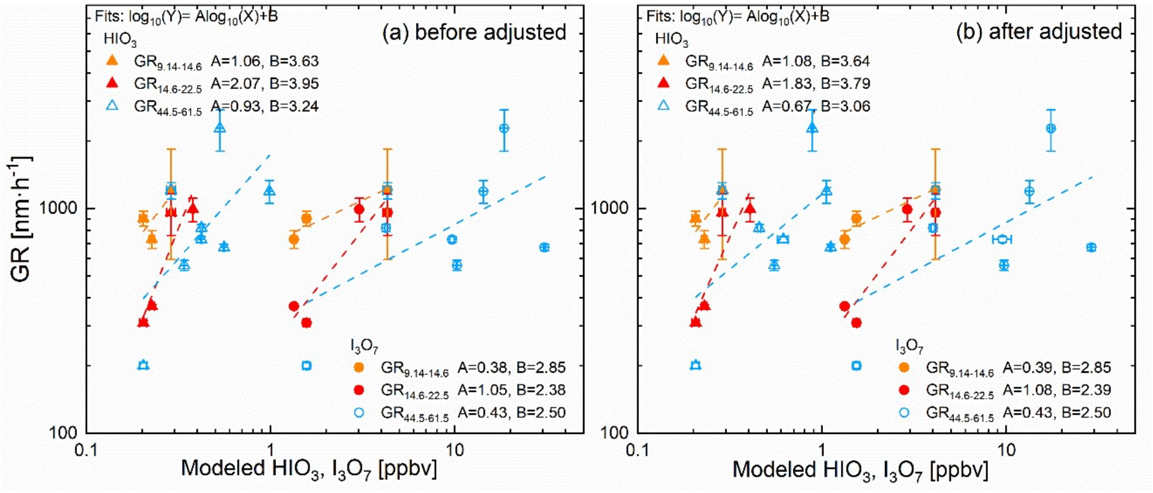

Figure S13. Growth rates of particles versus simulated HIO_3 and I_3O_7 mixing ratios before and after adjusting the original $I_2 + O_3$ branching ratio.

Table S1. Summary of the experimental conditions .

Experiments		I_2 (ppmv)	O_3 (ppmv)	IO (ppbv)	RH (%)	T (℃)
	E1-0	0	0	0		
	E1-1	1.321 ± 0.005	< DL	< DL		
	E1-2	1.335 ± 0.002	0.053 ± 0.003	0.23 ± 0.02		
E1	E1-3	1.357 ± 0.002	0.569 ± 0.003	0.29 ± 0.02	5.7 ± 0.4	17.1 ± 0.1
	E1-4	1.353 ± 0.001	4.218 ± 0.154	0.37 ± 0.03		
	E1-5	1.339 ± 0.003	9.396 ± 0.084	0.45 ± 0.01		
	E1-6	1.290 ± 0.001	29.333 ± 0.090	0.58 ± 0.01		
	E2-0	0	0	0		
	E2-1	0.808 ± 0.007	0	0		
	E2-2	0.787 ± 0.002	0.052 ± 0.002	0.21 ± 0.01		
E2	E2-3	0.778 ± 0.001	0.445 ± 0.003	0.21 ± 0.02	5.2 ± 0.2	17.7 ± 0.2
	E2-4	0.758 ± 0.002	4.244 ± 0.056	0.29 ± 0.01		
	E2-5	0.730 ± 0.001	9.245 ± 0.090	0.33 ± 0.02		
	E2-6	0.673 ± 0.002	28.898 ± 0.146	0.33 ± 0.01		
	E3-0	0	0	0		
	E3-1	6.967 ± 0.025	0	0		
	E3-2	6.933 ± 0.007	0.056 ± 0.003	0.39 ± 0.02		
E3	E3-3	7.039 ± 0.005	0.445 ± 0.003	0.61 ± 0.01	5.4 ± 0.6	18.4 ± 0.2
	E3-4	6.982 ± 0.003	4.295 ± 0.070	0.61 ± 0.02		
	E3-5	6.727 ± 0.003	9.009 ± 0.064	0.76 ± 0.02		
	E3-6	6.327 ± 0.004	29.472 ± 0.101	0.90 ± 0.02		

Table S2. Spectral fitting configurations and detection limits of trace gases in IO generation.

Trace gas	Fitting window (nm)	Interferences	Detection limits (ppbv)
IO	426.3-447.9	NO_2, H_2O	0.12

I_2	532.4-547.3	OIO, H_2O	7.5
O_3	256.2-273.9	/	5.6

Table S3. Gas-phase iodine chemistry scheme in F0AM: Bimolecular and thermal decomposition reactions (Rate constants without temperature dependence are given as recommended values for the temperature range 294–298 K).

Reaction	k (cm^3 $molecule^{-1}$ s^{-1})	Notes
$I_2 + O \rightarrow IO + I$	1.3×10^{-10}	7
$I_2 + O_3 \rightarrow IO + I + O_2$ [a]	$0.5 \times 10^{-10} \times \exp(-2050/T)$	5,6
$I_2 + O_3 \rightarrow IO + OIO$ [a]	$3.5 \times 10^{-10} \times \exp(-2050/T)$	
$I_2 + OH \rightarrow HOI + I$	1.8×10^{-10}	7
$I + O_3 \rightarrow IO + O_2$	$2.0 \times 10^{-11} \times \exp(-830/T)$	7
$I + HO_2 \rightarrow HI + O_2$	$1.5 \times 10^{-11} \times \exp(-1090/T)$	7
$I + I_2O \rightarrow I_2 + IO$	3.82×10^{-11}	17
$I + IO \rightarrow I_2O$	1.69×10^{-10}	18
$IO + HO_2 \rightarrow HOI + O_2$	$1.3 \times 10^{-11} \times \exp(-570/T)$	7
$IO + IO \rightarrow I + OIO$	$2.13 \times 10^{-11} \times \exp(-180/T) \times (1+\exp(-p/19142))$	19
$IO + IO \rightarrow I_2O_2$	$3.27 \times 10^{-11} \times \exp(-180/T) \times (1-0.65 \times \exp(-p/19142))$	19
$IO + OIO \rightarrow I_2O_3$	b	19
$IO + O_3 \rightarrow OIO + O_2$	3.6×10^{-16}	20
$IO + O \rightarrow I + O_2$	1.4×10^{-10}	7
$IO + OH \rightarrow HO_2 + I$	1.0×10^{-10}	21
$IO + I_2O_2 \rightarrow I_2O + OIO$	5.0×10^{-12}	22
$IO + I_2O_4 \rightarrow I_3O_5$	3.0×10^{-11}	22
$OIO + OIO \rightarrow I_2O_4$	c	19
$HI + OH \rightarrow I + H_2O$	3.0×10^{-11}	7
$HOI + OH \rightarrow IO + H_2O$	2.0×10^{-13}	23

$I_2O_2 + O_3 \rightarrow I_2O_3 + O_2$	4.0×10^{-14}	22
$I_2O_3 + O_3 \rightarrow I_2O_4 + O_2$	8.0×10^{-14}	22
$I_2O_2 + I_2O_2 \rightarrow I_2O_3 + I_2O$	1.0×10^{-11}	22
$I_2O_2 + OIO \rightarrow I_3O_4$	3.0×10^{-12}	22
$I_2O_2 + OIO \rightarrow I_2O_3 + IO$	1.0×10^{-11}	22
$I_3O_4 + O_3 \rightarrow I_3O_5 + O_2$	8.0×10^{-14}	22
$I_3O_5 + O_3 \rightarrow I_3O_6 + O_2$	1.0×10^{-13}	22
$I_3O_6 + O_3 \rightarrow I_3O_7 + O_2$	1.0×10^{-13}	22
$I_2O_2 + O_3 \rightarrow IOIO_4$	$8.2 \times 10^{-15} \times \exp(763/T)$	4
$IOIO_4 + H_2O \rightarrow HIO_3 + HOI + O_2$	$2.5 \times 10^{-12} \times \exp(-2481/T)$	4
$I_2O_2 \rightarrow IO + IO$	d	19
$I_2O_2 \rightarrow OIO + I$	$8.4 \times 10^{13} \times \exp(-12026/T)$	4
$I_2O_4 \rightarrow OIO + OIO$	e	19

[a] branching changed was listed in box model description

[b] $k = (4.687 \times 10^{-10} - 1.3855 \times 10^{-5} \times \exp(-0.75 \times p / 162.265) + 5.51868 \times 10^{-10} \times \exp(-0.75 \times p / 19932.8)) \times \exp(-3.31 \times 10^{-3} - 5.14 \times 10^{-3} \times \exp((-0.75 \times p / 32568.711) - 4.44\times 10^{-3} \times \exp(-0.75 \times p / 4081.609)) \times T)$

[c] $k = (1.1659 \times 10^{-9} - 7.79644 \times 10^{-10} \times \exp(-0.75 \times p / 2209.281) + 1.03779 \times 10^{-9} \times \exp(-0.75 \times p / 56815.381)) \times \exp(-8.13 \times 10^{-3} - 3.82 \times 10^{-3} \times \exp((-0.75 \times p / 4557.591) - 6.43 \times 10^{-3} \times \exp(-0.75 \times p / 41795.061)) \times T)$

[d] $k = (2.55355 \times 10^{11} - 4.41888 \times 10^{7} \times 0.75 \times p + 856.186 \times (0.75 \times p)^2 + 1.421881 \times 10^{-2} \times (0.75 \times p)^3) \times \exp((-11466.82304 + 597.01334 \times \exp(-0.75 \times p / 138262.325) - 167.339 \times \exp(-0.75 \times p / 4375.089)) / T)$

[e] $k = (-1.92626 \times 10^{14} + 4.67414 \times 10^{11} \times 0.75 \times p - 36865.1 \times (0.75 \times p)^2 - 3.09109 \times 10^{-2} \times (0.75 \times p)^3) \times \exp((-12302.15294 + 152.78367 \times \exp(-0.75 \times p / 4612.733) + 437.62868 \times \exp(-0.75 \times p / 42844.13)) / T)$

Table S4. Photolysis reactions of iodine chemistry in F0AM.

Reaction	j (s^{-1})
$I_2 + hv \rightarrow I + I$	1.8×10^{-4}
$HOI + hv \rightarrow I + OH$	5.8×10^{-6}
$IO + hv \rightarrow I + O$	2.2×10^{-4}
$OIO + hv \rightarrow I + O_2$	3.8×10^{-4}
$I_2O_2 + hv \rightarrow I + OIO$	5.4×10^{-5}
$I_2O_3 + hv \rightarrow IO + OIO$	1.4×10^{-5}
$I_2O_4 + hv \rightarrow OIO + OIO$	1.7×10^{-5}

Table S5. Heterogeneous reactions of iodine chemistry in F0AM

Species	Reactive uptake	Notes
IO	$\gamma = 0.02$	24
OIO	$\gamma = 1.0$	24
HI	$\gamma = 0.1$	25
HOI	$\gamma = 0.1$	26
I_2O_2	$\gamma = 0.02$	25
I_2O_3	$\gamma = 0.02$	25
I_2O_4	$\gamma = 0.02$	25
$IOIO_4$	$\gamma = 0.02$	a
HIO_3	$\gamma = 0.2$	a
I_3O_4	$\gamma = 0.02$	a
I_3O_5	$\gamma = 0.02$	a
I_3O_6	$\gamma = 0.02$	a
I_3O_7	$\gamma = 0.02$	a

[a] assumed

References

1 U. Platt and J. Stutz, *Differential Absorption Spectroscopy*, Springer, Berlin, 2008.

2 S. Coburn, B. Dix, R. Sinreich and R. Volkamer, *Atmos. Meas. Tech.*, 2011, **4**, 2421-2439.

3 J. Stutz and U. Platt, *Appl. Opt.*, 1996, **35**, 6041-6053.

4 H. Finkenzeller, S. Iyer, X.-C. He, M. Simon, T. K. Koenig, C. F. Lee, R. Valiev, V. Hofbauer, A. Amorim, R. Baalbaki, A. Baccarini, L. Beck, D. M. Bell, L. Caudillo, D. Chen, R. Chiu, B. Chu, L. Dada, J. Duplissy, M. Heinritzi, D. Kemppainen, C. Kim, J. Krechmer, A. Kürten, A. Kvashnin, H. Lamkaddam, C. P. Lee, K. Lehtipalo, Z. Li, V. Makhmutov, H. E. Manninen, G. Marie, R. Marten, R. L. Mauldin, B. Mentler, T. Müller, T. Petäjä, M. Philippov, A. Ranjithkumar, B. Rörup, J. Shen, D. Stolzenburg, C. Tauber, Y. J. Tham, A. Tomé, M. Vazquez-Pufleau, A. C. Wagner, D. S. Wang, M. Wang, Y. Wang, S. K. Weber, W. Nie, Y. Wu, M. Xiao, Q. Ye, M. Zauner-Wieczorek, A. Hansel, U. Baltensperger, J. Brioude, J. Curtius, N. M. Donahue, I. E. Haddad, R. C. Flagan, M. Kulmala, J. Kirkby, M. Sipilä, D. R. Worsnop, T. Kurten, M. Rissanen and R. Volkamer, *Nat. Chem.*, 2023, **15**, 129-135.

5 O. Gálvez, J. C. Gómez Martín, P. C. Gómez, A. Saiz-Lopez and L. F. Pacios, *Phys. Chem. Chem. Phys.*, 2013, **15**, 15572-15583.

6 A. C. Vikis and R. MacFarlane, *J. Phys. Chem.*, 1985, **89**, 812-815.

7 J. B. Burkholder, S. P. Sander, J. P. D. Abbatt, J. R. Barker, C. Cappa, J. D. Crounse, T. S. Dibble, R. E. Huie, C. E. Kolb and M. J. Kurylo, *Chemical kinetics and photochemical data for use in atmospheric studies; evaluation number 19*, Jet Propulsion Laboratory, Pasadena, CA, 2019.

8 T. R. Lewis, J. C. Gómez Martín, M. A. Blitz, C. A. Cuevas, J. M. C. Plane and A. Saiz-Lopez, *Atmos. Chem. Phys.*, 2020, **20**, 10865-10887.

9 K. L. Furneaux, L. K. Whalley, D. E. Heard, H. M. Atkinson, W. J. Bloss, M. J. Flynn, M. W. Gallagher, T. Ingham, L. Kramer, J. D. Lee, R. Leigh, G. B. McFiggans, A. S. Mahajan, P. S. Monks, H. Oetjen, J. M. C. Plane and J. D. Whitehead, *Atmos. Chem. Phys.*, 2010, **10**, 3645-3663.

10 X.-C. He, M. Simon, S. Iyer, H.-B. Xie, B. Rörup, J. Shen, H. Finkenzeller, D. Stolzenburg, R. Zhang, A. Baccarini, Y. J. Tham, M. Wang, S. Amanatidis, A. A. Piedehierro, A. Amorim, R. Baalbaki, Z. Brasseur, L. Caudillo, B. Chu, L. Dada, J. Duplissy, I. El Haddad, R. C. Flagan, M. Granzin, A. Hansel, M. Heinritzi, V. Hofbauer, T. Jokinen, D. Kemppainen, W. Kong, J. Krechmer, A. Kürten, H. Lamkaddam, B. Lopez, F. Ma, N. G. A. Mahfouz, V. Makhmutov, H. E. Manninen, G. Marie, R. Marten, D. Massabò, R. L. Mauldin, B. Mentler, A. Onnela, T. Petäjä, J. Pfeifer, M. Philippov, A. Ranjithkumar, M. P. Rissanen, S. Schobesberger, W. Scholz, B. Schulze, M. Surdu, R. C. Thakur, A. Tomé, A. C. Wagner, D. Wang, Y. Wang, S. K. Weber, A. Welti, P. M. Winkler, M. Zauner-Wieczorek, U. Baltensperger, J. Curtius, T. Kurtén, D. R. Worsnop, R. Volkamer, K. Lehtipalo, J. Kirkby, N. M. Donahue, M. Sipilä and M. Kulmala, *Science*, 2023, **382**, 1308-1314.

11 D. Stolzenburg, L. Fischer, A. L. Vogel, M. Heinritzi, M. Schervish, M. Simon, A. C. Wagner, L. Dada, L. R. Ahonen, A. Amorim, A. Baccarini, P. S. Bauer, B. Baumgartner, A. Bergen, F. Bianchi, M. Breitenlechner, S. Brilke, S. Buenrostro Mazon, D. Chen, A. Dias, D. C. Draper, J. Duplissy, I. El Haddad, H. Finkenzeller, C. Frege, C. Fuchs, O. Garmash, H. Gordon, X. He, J. Helm, V. Hofbauer, C. R. Hoyle, C. Kim, J. Kirkby, J. Kontkanen, A. Kürten, J. Lampilahti, M. Lawler, K. Lehtipalo, M. Leiminger, H. Mai, S. Mathot, B. Mentler, U. Molteni, W. Nie, T. Nieminen, J. B. Nowak, A. Ojdanic, A. Onnela, M. Passananti, T. Petäjä, L. L. J. Quéléver, M. P. Rissanen, N. Sarnela, S. Schallhart, C. Tauber, A. Tomé,

R. Wagner, M. Wang, L. Weitz, D. Wimmer, M. Xiao, C. Yan, P. Ye, Q. Zha, U. Baltensperger, J. Curtius, J. Dommen, R. C. Flagan, M. Kulmala, J. N. Smith, D. R. Worsnop, A. Hansel, N. M. Donahue and P. M. Winkler, *Proc. Natl. Acad. Sci. U. S. A.*, 2018, **115**, 9122-9127.

12 X.-C. He, Y. J. Tham, L. Dada, M. Wang, H. Finkenzeller, D. Stolzenburg, S. Iyer, M. Simon, A. Kürten, J. Shen, B. Rörup, M. Rissanen, S. Schobesberger, R. Baalbaki, D. S. Wang, T. K. Koenig, T. Jokinen, N. Sarnela, L. J. Beck, J. Almeida, S. Amanatidis, A. Amorim, F. Ataei, A. Baccarini, B. Bertozzi, F. Bianchi, S. Brilke, L. Caudillo, D. Chen, R. Chiu, B. Chu, A. Dias, A. Ding, J. Dommen, J. Duplissy, I. El Haddad, L. Gonzalez Carracedo, M. Granzin, A. Hansel, M. Heinritzi, V. Hofbauer, H. Junninen, J. Kangasluoma, D. Kemppainen, C. Kim, W. Kong, J. E. Krechmer, A. Kvashin, T. Laitinen, H. Lamkaddam, C. P. Lee, K. Lehtipalo, M. Leiminger, Z. Li, V. Makhmutov, H. E. Manninen, G. Marie, R. Marten, S. Mathot, R. L. Mauldin, B. Mentler, O. Möhler, T. Müller, W. Nie, A. Onnela, T. Petäjä, J. Pfeifer, M. Philippov, A. Ranjithkumar, A. Saiz-Lopez, I. Salma, W. Scholz, S. Schuchmann, B. Schulze, G. Steiner, Y. Stozhkov, C. Tauber, A. Tomé, R. C. Thakur, O. Väisänen, M. Vazquez-Pufleau, A. C. Wagner, Y. Wang, S. K. Weber, P. M. Winkler, Y. Wu, M. Xiao, C. Yan, Q. Ye, A. Ylisirniö, M. Zauner-Wieczorek, Q. Zha, P. Zhou, R. C. Flagan, J. Curtius, U. Baltensperger, M. Kulmala, V.-M. Kerminen, T. Kurtén, N. M. Donahue, R. Volkamer, J. Kirkby, D. R. Worsnop and M. Sipilä, *Science*, 2021, **371**, 589-595.

13 L. Dada, K. Lehtipalo, J. Kontkanen, T. Nieminen, R. Baalbaki, L. Ahonen, J. Duplissy, C. Yan, B. Chu, T. Petäjä, K. Lehtinen, V.-M. Kerminen, M. Kulmala and J. Kangasluoma, *Nat. Protoc.*, 2020, **15**, 1013-1040.

14 K. Lehtipalo, J. Leppä, J. Kontkanen, J. Kangasluoma, A. Franchin, D. Wimnner, S. Schobesberger, H. Junninen, T. Petäjä and M. Sipilä, *Boreal Environ. Res.*, 2014, **19**, 215-236.

15 D. Li, W. Nie, Y. Liu, C. Yan, D. Ge, Q. Zha, C. Liu, J. Wang, J. Wang, L. Wang, T. Liu, X. Chi and A. Ding, *Environ. Sci. Technol. Lett.*, 2024, **11**, 709-715.

16 Y. Zhang, D. Li, X. C. He, W. Nie, C. Deng, R. Cai, Y. Liu, Y. Guo, C. Liu, Y. Li, L. Chen, Y. Li, C. Hua, T. Liu, Z. Wang, J. Xie, L. Wang, T. Petäjä, F. Bianchi, X. Qi, X. Chi, P. Paasonen, Y. Liu, C. Yan, J. Jiang, A. Ding and M. Kulmala, *Atmos. Chem. Phys.*, 2024, **24**, 1873-1893.

17 C. Fortin, V. Fèvre-Nollet, F. Cousin, P. Lebègue and F. Louis, *Atmos. Environ.*, 2019, **214**, 116838.

18 W. J. Bloss, D. M. Rowley, R. A. Cox and R. L. Jones, *J. Phys. Chem. A*, 2001, **105**, 7840-7854.

19 A. Saiz-Lopez, R. P. Fernandez, C. Ordóñez, D. E. Kinnison, J. C. Gómez Martín, J. F. Lamarque and S. Tilmes, *Atmos. Chem. Phys.*, 2014, **14**, 13119-13143.

20 T. J. Dillon, M. E. Tucceri and J. N. Crowley, *Phys. Chem. Chem. Phys.*, 2006, **8**, 5185-5198.

21 H. Bösch, C. Camy-Peyret, M. P. Chipperfield, R. Fitzenberger, H. Harder, U. Platt and K. Pfeilsticker, *J. Geophys. Res.:Atmos.*, 2003, **108**.

22 J. C. Gómez Martín, T. R. Lewis, M. A. Blitz, J. M. C. Plane, M. Kumar, J. S. Francisco and A. Saiz-Lopez, *Nat. Commun.*, 2020, **11**, 4521.

23 G. McFiggans, J. M. C. Plane, B. J. Allan, L. J. Carpenter, H. Coe and C. O'Dowd, *J. Geophys. Res.:Atmos.*, 2000, **105**, 14371-14385.

24 A. Saiz-Lopez, J. M. C. Plane, A. S. Mahajan, P. S. Anderson, S. J. B. Bauguitte, A. E. Jones, H. K. Roscoe, R. A. Salmon, W. J. Bloss, J. D. Lee and D. E. Heard, *Atmos. Chem. Phys.*, 2008, **8**, 887-900.

25 T. Sherwen, J. A. Schmidt, M. J. Evans, L. J. Carpenter, K. Großmann, S. D. Eastham, D. J. Jacob, B.

Dix, T. K. Koenig, R. Sinreich, I. Ortega, R. Volkamer, A. Saiz-Lopez, C. Prados-Roman, A. S. Mahajan and C. Ordóñez, *Atmos. Chem. Phys.*, 2016, **16**, 12239-12271.

26 Y. J. Tham, X.-C. He, Q. Li, C. A. Cuevas, J. Shen, J. Kalliokoski, C. Yan, S. Iyer, T. Lehmusjärvi, S. Jang, R. C. Thakur, L. Beck, D. Kemppainen, M. Olin, N. Sarnela, J. Mikkilä, J. Hakala, M. Marbouti, L. Yao, H. Li, W. Huang, Y. Wang, D. Wimmer, Q. Zha, J. Virkanen, T. G. Spain, S. O'Doherty, T. Jokinen, F. Bianchi, T. Petäjä, D. R. Worsnop, R. L. Mauldin, J. Ovadnevaite, D. Ceburnis, N. M. Maier, M. Kulmala, C. O'Dowd, M. Dal Maso, A. Saiz-Lopez and M. Sipilä, *Proc. Natl. Acad. Sci. U. S. A.*, 2021, **118**, e2009951118.
